# Natural Cinnamic Acids, Synthetic Derivatives and Hybrids with Antimicrobial Activity

**DOI:** 10.3390/molecules191219292

**Published:** 2014-11-25

**Authors:** Juan David Guzman

**Affiliations:** Departamento de Química y Biología, División de Ciencias Básicas, Universidad del Norte, Km. 5 vía Puerto Colombia, Barranquilla 081007, Colombia; E-Mail: jguzmand@uninorte.edu.co; Tel.: +57-5-3509509 (ext. 3237); Fax: +57-5-3598852

**Keywords:** cinnamic acid, coumaric acids, hybrids, antimicrobial, tuberculosis

## Abstract

Antimicrobial natural preparations involving cinnamon, storax and propolis have been long used topically for treating infections. Cinnamic acids and related molecules are partly responsible for the therapeutic effects observed in these preparations. Most of the cinnamic acids, their esters, amides, aldehydes and alcohols, show significant growth inhibition against one or several bacterial and fungal species. Of particular interest is the potent antitubercular activity observed for some of these cinnamic derivatives, which may be amenable as future drugs for treating tuberculosis. This review intends to summarize the literature data on the antimicrobial activity of the natural cinnamic acids and related derivatives. In addition, selected hybrids between cinnamic acids and biologically active scaffolds with antimicrobial activity were also included. A comprehensive literature search was performed collating the minimum inhibitory concentration (MIC) of each cinnamic acid or derivative against the reported microorganisms. The MIC data allows the relative comparison between series of molecules and the derivation of structure-activity relationships.

## 1. Introduction

Cinnamic acids are a group of aromatic carboxylic acids (C_6_–C_3_) appearing naturally in the plant kingdom. They are formed in the biochemical route that yields lignin, the polymeric material that provides mechanical support to the plant cell wall [1]. Cinnamic acids occur in all green plants [2], although in minute quantities covalently bound to cell walls [3], but also in the reproductive organs of flowering plants [4]. Cinnamic acids are formed in the biosynthetic pathway leading to phenyl-propanoids, coumarins, lignans, isoflavonoids, flavonoids, stilbenes, aurones, anthocyanins, spermidines, and tannins [5]. These secondary metabolites play key physiological roles in plant growth, development, reproduction and disease resistance [6,7]. The first step of this pathway is catalyzed by the phenylalanine ammonia lyase (PAL), a widely distributed phenylpropanoid enzyme present in green plants, algae, fungi, and even in some prokaryotes [8]. This enzyme deaminates l-phenylalanine to yield (*E*)-cinnamic acid, which undergoes other enzymatic transformations, yielding a diversity of related products [5].

The term “cinnamic” derives from the spice cinnamon (*Cinnamomum zeilanicum*) which has been used since antiquity as a flavoring agent and for its stimulant, carminative, antiseptic and insecticide properties [9]. The bark of several species of *Cinnamomum* contain considerable amounts of (*E*)-cinnamaldehyde, a volatile aldehyde responsible for the pungent, sweet and hot flavor of cinnamon [10,11]. Cinnamaldehyde and the essential oils of the species of *Cinnamomum* have antimicrobial activity both against bacteria and fungi [12,13]. Cinnamic acids are also readily available from coffee beans, tea, mate, cocoa, apples and pears, berries, citrus, grape, brassicas vegetables, spinach, beetroot, artichoke, potato, tomato, celery, faba beans, and cereals [14]. Cinnamic acids often appear as ester conjugates with quinic acid, known as the chlorogenic acids, but they can also form esters with other acids, sugars or lipids, or form amides with aromatic and aliphatic amines.

**Figure 1:**
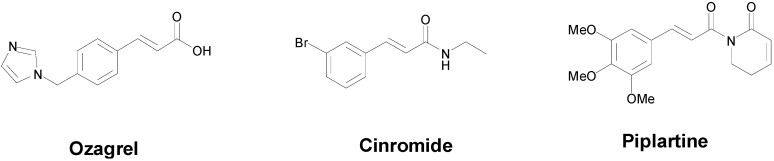
Chemical structures of some therapeutically important cinnamic acid-containing molecules: ozagrel, cinromide and piplartine.

In the last ten years, the interest of researchers on the cinnamic acid moiety has notably increased. The number of published reports having the word “cinnamic” in the title, has almost doubled, from 341 in the years 1993–2003 to 633 in the period 2004–2014 according to the Scopus database (until mid-November 2014). If both “cinnamic” and “antimicrobial” keywords are used, the number of published articles increased from 1 in the period 1993–2003, to 7 in the period 2004–2014. There is no doubt that the cinnamic acids currently attracts the attention of chemists from different perspectives. Ozagrel (Figure 1), a thromboxane A2 synthase inhibitor, is in fact an imidazole *para*-substituted cinnamic acid that is employed therapeutically for treating acute ischemic stroke [15]. Cinromide (Figure 1) is an antiepileptic experimental drug studied in clinical trials during the 80 decade with a favorable profile to suppress generalized convulsions, but however displayed considerable toxicity [16]. Piplartine (Figure 1) is another cinnamic-related molecule showing an attractive biological horizon [17]. This cinnamic amide was first-time isolated from the roots of *Piper tuberculatum* [18], and later proved to be a promising anti-cancer scaffold [19,20].

Several reviews and studies have appeared in the literature focusing on a particular medicinal application of cinnamic-related molecules, for example on anticancer [21], antituberculosis [22], antimalarial [23], antifungal [24], antimicrobial [25], antiatherogenic [26] and antioxidant [25] activities. In addition a number of reviews directed towards the synthetic methods used to prepare cinnamic acids and related molecules have appeared in the literature [27,28,29]. Cinnamic acids have also been used by medicinal chemists to alter the potency, permeability, solubility or other parameters of a selected drug or pharmacophore.

Infectious diseases caused by bacteria, fungi and viruses are still a prominent global health problem, particularly in developing, low-income countries [30,31]. Every year in the whole planet, around 4 million persons die from acute respiratory infections, around 3 million individuals die from enteric infections, around 1.8 persons die from human immunodeficiency virus (HIV), around 1.3 million die from tuberculosis (TB), and 0.7 million die from malaria [32,33,34]. Other thousand people deeply suffer the consequences of being infected by neglected tropical pathogens *Schistosoma mansoni*, *Onchocerca volvulus*, *Trypanosoma spp*., *Leishmania spp.*, *Mycobacterium ulcerans*, *Mycobacterium leprae*, *Wucheria spp*., and others. Although some of these diseases are caused by parasites, many are caused by bacteria and fungi. Anti-bacterial and anti-fungal drugs, which are broadly known as antimicrobials, started to be used in chemotherapy since the 1940 decade [35]. New antimicrobial classes were discovered in the 1940–1970 period, and were successfully introduced to clinical practice. However as soon as the new drugs were employed, the first drug-resistant strains started to appear [36,37]. In addition the widespread use of antibiotics for animal feed and human consumption during the last forty years, has fostered the emergence of resistance in several pathogenic organisms. Infection with drug-resistant strains is typically associated with longer treatment times, higher toxicity and higher costs. Nowadays some infections are extremely difficult to treat such as extensively drug-resistant tuberculosis (XDR-TB) [38], community-associated methicillin-resistant *Staphylococcus aureus* (MRSA) [39] and pan-resistant *Klebsiella* and *Escherichia coli* strains [40], and they pose an enormous challenge to clinicians. The presence of these “superbugs” call for drugs with novel mechanisms of action [41]. The cinnamic skeleton is considered an interesting scaffold for the development of novel antimicrobials, however little is known about its antimicrobial mechanism of action. A recent report proposed that cinnamic acids caused fungal growth inhibition by interacting with benzoate 4-hydroxylase, an enzyme responsible for aromatic detoxification [42]. However this enzyme occurs in fungi but not in prokaryotes, and because the cinnamic acids have proven anti-bacterialeffects [43,44,45,46], other targets may be implicated in their biological effects.

This review intends to bring the attention of scientists on the antimicrobial potential of the cinnamic acids. The focus is brought to the chemical entities containing the cinnamic skeleton, displaying fungal or bacterial growth inhibition. Emphasis is placed on whole-cell inhibitory potency, structure-activity relationships and mechanism of action studies. The review is organized in different sections according to the functional groups decorating the C_6_–C_3_ cinnamic skeleton. The first section deals with natural and synthetic cinnamic acids, the second section is concerned with cinnamic esters and amides, the third section deals with cinnamic aldehydes and alcohols, and finally the fourth section covers the hybrid covalently-bound molecules between a cinnamic acid and any other biologically-relevant molecule.

## 2. Natural and Synthetic Cinnamic Acids

Honey and propolis are both bee (*Apis mellifera*) products made from the nectar of the flowers, and they have long been used for their antimicrobial properties [47,48]. We should recall that a report from 1978, postulated the presence of cinnamic amides in the reproductive organs of flowers [4]. Propolis and honey contain hundreds of different organic compounds, and the cinnamic acids and their esters are typically present in these natural bees products [49,50,51,52]. Cinnamic acids with varied substitution on the aryl ring, and their esters have been identified in Iranian propolis showing minimum inhibitory concentration (MIC) values between 125 and 500 mg/L against bacteria and fungi [52]. Other studies have confirmed the antimicrobial potential of propolis [53,54]. Although secondary metabolites such as flavonoids, sesquiterpenoids present in propolis may have antimicrobial activity, cinnamic acids are likely to contribute to the observed effect.

The minimum inhibitory concentration (MIC) values for the natural cinnamic acids against different bacteria, as determined by different researchers using different methods, are shown in Table 1. It was surprising to find huge differences in the MIC values for the same compounds against the same species as reported by different authors. The existence of controversial results was already noted by Wen *et al.*, in the seminal work of the antilisterial effect of natural phenolic acids [46]. The differences may be attributed to the diversity of experimental methods for MIC determination, often measuring distinct end-points, using different inoculum sizes, different culture media, and using particular strains with varying susceptibilities [55]. Although these discrepancies obscure the antimicrobial potential of the cinnamic acid class, there is clear tendency of the molecules to inhibit the growth of a wide variety of microorganisms by molecular mechanisms that are still unknown.

**Figure 2:**
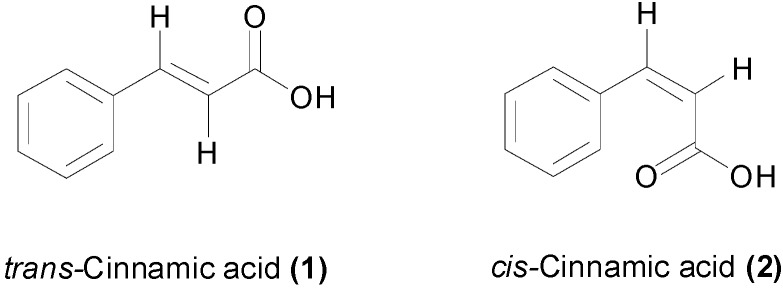
Chemical structures of *trans*- and *cis*-cinnamic acids

Cinnamic acid (**1**, Figure 2) showed a weak antibacterial effect against most of Gram-negative and Gram-positive species of bacteria, with MIC values higher than 5.0 mM [12,46,56,57,58] (Table 1). The same level of potency was observed against the fish pathogens *Aeromonas hydrophila*, *Aeromonas salmonicida* and *Edwardiella tarda* with MIC values between 5.6 and 7.7 mM [59]. However cinnamic acid was found to be much more active against the tuberculosis-causing bacteria, *Mycobacterium tuberculosis* H_37_Rv, with an MIC values of 270–675 µM using the SPOTi and the radiometric Bactec assays [60,61,62]. The free carboxylic acid and the presence of the α,β-unsaturation were both required for the anti-TB activity [60]. Rastogi *et al.*, reported an MIC value of 675 µM against the H_37_Rv strain and varying values between 337 µM and 1.4 mM for multiple drug-resistant (MDR) clinical *M. tuberculosis* isolates [62]. The study found that **1** enhanced synergistically the effect of anti-TB drugs such as amikacin, ofloxacin and clofazimine. In addition its geometric isomer, *cis*-cinnamic acid (**2**) (Figure 2), was approximately 120 times more active than the *trans* isomer, with minimum bactericidal concentrations (MBC) values of 16.9 µM for **2**, compared to 2.0 mM for **1**, against an MDR *M. tuberculosis* strain [63]. The specific anti-TB effect of cinnamic acid may explain the traditional use of storax (*Liquidambar orientalis*) and cinnamon for treating TB in the 19^th^ century [64]. Cinnamic acid also demonstrated anti-fungal activity with MIC values of 1.7 mM against *Aspergillus terreus* and *Aspergillus flavus*, being more active against *Aspergillus niger* with an MIC value of 844 µM [65]. Against *Candida albicans*, an MIC value of 405 µM has been found [66], which is comparable to the potency against *M. tuberculosis*.

**Figure 3:**
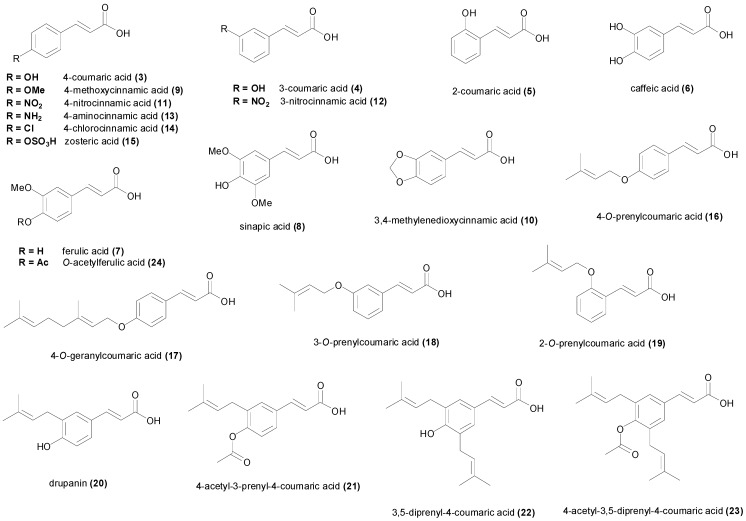
Chemical structures of differently substituted natural and synthetic cinnamic acids.

The widely distributed natural phenol 4-hydroxycinnamic acid (**3**, Figure 3), also known as 4-coumaric acid, has been found to be comparatively more potent bacterial growth inhibitor compared to cinnamic acid (**1**) (Table 1). However the reported MIC values of 4-coumaric acid vary to great extent from one species or strain to another. The MIC values against some Gram-negative bacteria such as *Shigella disenteriae* 51302 was low (MIC = 61 µM) [67], but however against *Neisseria gonorrhoeae* or *Listeria monocytogenes* or *E. coli*, the MIC was high (MIC > 6.0 mM) [46,58]. The same was true against Gram-positive species, with some studies reporting low MIC values for strains of *Staphylococcus aureus* and *Bacillus subtilis* whereas other reports have published higher MIC values [67,68,69,70]. A study showed that **3** disrupted the outer membrane of the Gram-negative bacteria *S. disenteriae* increasing the permeabilization, and in addition, the compound interacted with DNA and thus it may inhibit essential biochemical processes related to nucleic acids [67]. 4-Coumaric acid (**3**) completely inhibited *M. tuberculosis* H_37_Rv growth at 244 µM concentration [60], being therefore slightly more active than cinnamic acid. The compound had weak inhibition of lactic acid bacteria with MIC values of 6.09 mM [71,72] but was however relatively more active against the economically important phytopathogenic fungi *Fusarium oxysporum* and *Fusarium verticillioides*, with MIC values of 3.5 mM and 2.2 mM respectively [73]. Against *Aspergillus spp* MIC values have been reported between 1.5 mM and 760 µM [65].

The structural isomers 3-coumaric acid (**4**) and 2-coumaric acid (**5**) (Figure 3 and Table 1) are less common in nature [74,75] compared to the 4-isomer, and have been less studied. In particular, reports of the antimicrobial activity of the 3-coumaric acid are scarce. 3-Coumaric acid (**4**) was less active (MIC = 366 µM) compared with the 4-isomer against *M. tuberculosis* H_37_Rv, while 2-coumaric acid (**5**) was the isomer with the highest activity (MIC = 122 µM) [60]. Similarly, 2-coumaric acid showed stronger antimicrobial activity against *E. coli*, *S. aureus*, *Salmonella typhimurium* and *Lactobacillus rhamnosus* in comparison with 4-coumaric acid, with MIC values between 1.5 mM and 760 µM [69]. However another report published the same MIC values against *S. aureus* (MIC > 3.6 mM), *Bacillus cereus* (MIC = 2.4 mM), *E. coli* (MIC = 2.7 mM) and *S. typhimurium* (MIC = 2.7 mM) [70]. The 3-isomer (**4**) displayed moderate antifungal activity (MIC > 1.5 mM) against *A. terreus*, *A. flavus* and *A. niger* [65].

The other abundant cinnamic acids in nature are caffeic acid (**6**), ferulic acid (**7**) and sinapic acid (**8**) (Figure 3), which have been studied for their antimicrobial activities [58,68,73]. A similar pattern was observed for these three natural cinnamic acids, showing a weak growth inhibition against Gram-negative bacteria compared to Gram-positive bacteria and fungi (Table 1). The pH of the media has been reported to exert influence on growth inhibition, with lower pH values increasing the activity of the acids [76] by probably favoring a greater proportion of un-dissociated acid. The lowest MIC values for caffeic acid (**6**) were found against some strains of *S. aureus* and *Streptococcus pyogenes* 10535 (MIC = 694 µM) [69,77]. Caffeic acid showed significant growth inhibition of planktonic *C. albicans* with MIC between 694 and 710 µM [77,78]. Other species of bacteria and fungi were less susceptible to caffeic acid. Ferulic acid (**7**) demonstrated significant antibacterial activity against *S. aureus* 209 and *Streptococcus pyogenes* 10535 with MIC values of 644 µM [77]. *Pseudomonas aeruginosa* ATCC 10145 and *E. coli* CECT 434 were also susceptible to ferulic acid (MIC = 515 µM) [79]. In addition the strain *A. niger* ATCC 11394 showed a low MIC value (MIC = 322 µM) [65], however the MIC value against another strain of *A. niger* was found to be higher than 10 mM [80]. Interestingly *A. flavus* UBA 294 was the microorganism with the lowest MIC value for ferulic acid (MIC = 161 µM) [65]. A similar pattern was noticed for *C. albicans*, one strain being reported as susceptible with an MIC value of 659 µM [81], whereas another report showed an MIC value higher than 10 mM [80]. Sinapic acid (**8**), which is the most substituted of the common naturally-occurring cinnamic acids, showed significant activity against *S. aureus* 209 and *Streptococcus pyogenes* 10535 (MIC = 558 µM) [77]. The acid also demonstrated anti-*Campylobacter* activity with MIC values ranging from 696 µM to 1.40 mM [82]. Sinapic acid was fairly active against *L. monocytogenes* ATCC 7644 with an MIC value of 900 µM [83]. Sinapic acid (**8**) was completely inactive at a concentration of 4.46 mM against the phytopathogenic fungi *F. oxysporum*, *A. flavus*, *Penicillium brevicompactum* and others [73], in contrast with caffeic and ferulic acid.

Some cinnamic acids with a particular substitution pattern on the aryl ring have been prepared and examined for their antimicrobial activity. The compound 4-methoxycinnamic acid (**9**) was isolated from the Argentinian medicinal plant *Baccharis grisebachii* and its antimicrobial activity was evaluated [84]. This acid showed a potent antibacterial and antifungal effect with MIC values ranging between 50.4 and 449 µM (Table 1) [44,84]. Interestingly, the acid (**9**) showed higher growth inhibition against fungal species compared to bacteria, and Gram-negative and Gram-positive bacteria were equally inhibited by the compound. The acid 3,4-methylenedioxycinnamic acid (**10**) has been reported to inhibit *Mycobacterium tuberculosis* H_37_Rv with one report displaying an MIC value of 312 µM [60] and the other an MIC value higher than 520 µM [85]. The effect of the position of the nitro group on antimicrobial activity suggest that 4-nitrocinnamic acid (**11**) is more active than 3-nitrocinnamic acid (**12**), however the comparison data results from two different studies [44,86]. The seminal report from 1940, found that none of the positional isomers of nitrocinnamic acid inhibited *S. aureus* or *E. coli* at the highest dilution tested [87]. A noteworthy MIC value was found for **12** against the fungal species *A. niger* and *C. albicans* (MIC = 43.5 µM) [44]. Although the nitro groups can be readily reduced to amino groups, only 4-aminocinnamic (**13**) acid has been evaluated for its antimicrobial properties. This acid showed inhibitory activity of *B. subtilis* and *E. coli* with respective MIC values of 602 and 708 µM [86]. No information on the antimicrobial properties of the positional isomers of 4-aminocinnamic acid could be found. Although most of the halogen derivatives of cinnamic acid have been prepared [88,89], no information about their antimicrobial profile of activity was found in literature, except for 4-chlorocinnamic acid (**14**). This acid showed MIC values of 708 µM against both *E. coli* and *B. subtilis* [86]. Zosteric acid (**15**) which is naturally present in the eelgrass *Zostera marina*, has been found to display powerful antifouling properties by preventing bacterial biofilm formation on the surface of water-submerged objects [90]. Zosteric acid did not show any growth inhibitory activity against *M. tuberculosis* H_37_Rv [60], however the compound inhibited biofilm formation of *C. albicans* at 41 µM [91], but no MIC values were found in the literature search.

Among the prenylated coumaric acids, 4-*O*-prenylcoumaric acid (**16**) showed potent inhibition of *M. tuberculosis* H_37_Rv with an MIC value of 86 µM [60]. The MIC values for 4-*O*-geranylcoumaric acid (**17**), 3-*O*-prenylcoumaric acid (**18**) and 2-*O*-prenylcoumaric acid (**19**) against the same bacterial species were found to be respectively 69, 172 and 258 µM [60]. If we compare the anti-TB activity of the coumaric acids with the *O*-prenylcoumaric acids, it is clear that *O*-prenylation increases the activity for the 4- and 3-isomers, whereas for the 2-isomer, *O*-prenylation reduces the anti-TB activity. The *C*-prenylated coumaric acid drupanin (**20**), also known as 3-prenyl-4-coumaric acid, showed fungal growth inhibition particularly against dermatophytes species such as *Epidermophyton floccosum* C114, *Microsporum gypseum* C115, *Microsporum canis* C112, *Trichophyton mentagrophytes* ATCC 9972 and *Trichophyton rubrum* C113 with respective MIC values of 215, 431, 431, 431 and 431 µM [92]. Against the bacterial species, drupanin was weakly active with MIC values higher than 1.1 mM. The *O*-acetyl derivative of drupanin, 4-acetyl-3-prenyl-4-coumaric acid (**21**) showed a similar pattern of activity, being able to inhibit the dermatophyte *E. floccosum* C114 with an MIC value of 364 µM [92]. It was therefore slightly less active than the non-acetylated compound.

**Table 1:** Minimum inhibitory concentration values of natural and synthetic cinnamic acids **1**–**24**.

Compound	Microbial Strain	MIC	Refs.
cinnamic acid (**1**)	*Aeromonas hydrophila* MTCC 646	7.7 mM	[59]
	*Aeromonas salmonicida* MTCC 1522	5.6 mM	[59]
	*Aspergillus flavus* UBA294	1.7 mM	[65]
	*Aspergillus niger* ATCC 11394	844 µM	[65]
	*Aspergillus terreus* INM 031783	1.7 mM	[65]
	*Candida albicans*	405 µM	[66]
	*Edwardiella tarda* MTCC 2400	7.0 mM	[59]
	*Enterococcus faecalis*	6.75 mM	[12]
	*Escherichia coli*	6.75 mM	[12]
	*Escherichia coli*	5.0 mM	[56]
	*Escherichia coli*	>6.75 mM	[58]
	*Escherichia coli* ATCC 25922	9.0 mM	[57]
	*Listeria monocytogenes*	13.5 mM	[46]
	*Morganella morganni*	>6.75 mM	[58]
	*Mycobacterium tuberculosis* H_37_Rv	270 µM	[60]
	*Mycobacterium tuberculosis* H_37_Rv	675 µM	[62]
	*Neisseria gonorrhoeae*	6.75 mM	[58]
	*Pasteurella multocida*	>6.75 mM	[58]
	*Proteus mirabilis*	>6.75 mM	[58]
	*Pseudomonas aeruginosa*	6.75 mM	[12]
	*Salmonella sp.*	6.75 mM	[12]
	*Salmonella typhimurium* LT2	7.5 mM	[56]
	*Staphylococcus aureus*	6.75 mM	[12]
	*Staphylococcus epidermis*	6.75 mM	[12]
	*Streptococcus pyogenes* 10535	844 µM	[77]
	*Vibrio parahaemolyticus*	6.75 mM	[12]
4-coumaric acid (**3**)	*Aspergillus flavus* UBA294	1.5 mM	[65]
	*Aspergillus niger* ATCC 11394	761 µM	[65]
	*Aspergillus terreus* INM 031783	1.5 mM	[65]
	*Bacillus cereus* No-8	2.44 mM	[70]
	*Bacillus subtilis* NCIMB 8649	2.0 mM	[68]
	*Bacillus subtilis* 9372	122 µM	[67]
	*Escherichia coli* NCIMB 12210	2.0 mM	[68]
	*Escherichia coli*	6.09 mM	[58]
	*Escherichia coli* #916	3.04 mM	[69]
	*Escherichia coli* O157:H7	2.74 mM	[70]
	*Escherichia coli* ATCC 25922	490 µM	[67]
	*Fusarium oxysporum*	3.5 mM	[73]
	*Fusarium verticillioides*	2.2. mM	[73]
	*Lactobacillus brevis*	6.09 mM	[72]
	*Lactobacillus collinoides*	6.09 mM	[72]
	*Lactobacillus hilgardii* IFI-CA 49	6.09 mM	[71]
	*Lactobacillus rhamnosus* #299	3.04 mM	[69]
	*Listeria monocytogenes*	13.4 mM	[46]
	*Morganella morganni*	>6.09 mM	[58]
	*Mycobacterium tuberculosis* H_37_Rv	244 µM	[60]
	*Neisseria gonorrhoeae*	6.09 mM	[58]
	*Pasteurella multocida*	6.09 mM	[58]
	*Pediococcus pentosaceus* IFI-CA 85	4.87 mM	[71]
	*Proteus mirabilis*	>6.09 mM	[58]
	*Pseudomonas syringae* NCIMB 649	2.0 mM	[68]
	*Saccharomyces cerevisiae* 019 391	>8.0 mM	[68]
	*Salmonella typhimurium* #450	3.04 mM	[69]
	*Salmonella typhimurium* NRRL E4463	2.44 mM	[70]
	*Salmonella typhimurium* 50013	122 µM	[67]
	*Schizosaccharomyces pombe* 039 917	8.0 mM	[68]
	*Shigella disenteriae* 51302	61 µM	[67]
	*Sporobolomyces roseus* 043 529	8.0 mM	[68]
	*Staphylococcus aureus* # 917	761 µM	[69]
	*Staphylococcus aureus* NCTC 10657	>3.65 mM	[70]
	*Staphylococcus aureus* 6538	122 µM	[67]
	*Streptococcus pneumoniae* ATCC 49619	122 µM	[67]
	*Streptococcus pyogenes* 10535	761 µM	[77]
3-coumaric acid (**4**)	*Aspergillus flavus* UBA294	1.5 mM	[65]
	*Aspergillus niger* ATCC 11394	1.5 mM	[65]
	*Aspergillus terreus* INM 031783	1.5 mM	[65]
	*Mycobacterium tuberculosis* H_37_Rv	366 µM	[60]
2-coumaric acid (**5**)	*Bacillus cereus* No-8	2.44 mM	[70]
	*Escherichia coli* #916	1.5 mM	[69]
	*Escherichia coli* O157:H7	2.74 mM	[70]
	*Lactobacillus rhamnosus* #299	1.5 mM	[69]
	*Morganella morganni*	>6.09 mM	[58]
	*Mycobacterium tuberculosis* H_37_Rv	122 µM	[60]
	*Neisseria gonorrhoeae*	>6.09 mM	[58]
	*Pasteurella multocida*	>6.09 mM	[58]
	*Proteus mirabilis*	>6.09 mM	[58]
	*Salmonella typhimurium* NRRL E4463	2.44 mM	[70]
	*Salmonella typhimurium* #450	1.5 mM	[69]
	*Staphylococcus aureus* NCTC 10657	>3.65 mM	[70]
	*Staphylococcus aureus* # 917	760 µM	[69]
caffeic acid (**6**)	*Aspergillus flavus* UBA294	>1.39 mM	[65]
	*Aspergillus flavus*	>5.5 mM	[73]
	*Aspergillus fumigatus*	>5.5 mM	[73]
	*Aspergillus niger* ATCC 11394	>1.39 mM	[65]
	*Aspergillus terreus* INM 031783	>1.39 mM	[65]
	*Bacillus cereus* No-8	1.94 mM	[70]
	*Bacillus subtilis* NCIMB 8649	4.0 mM	[68]
	*Campylobacter jejuni* KC40	>1.0 mM	[93]
	*Candida albicans* 62	694 µM	[77]
	*Candida albicans* biofilm	1.42 mM	[78]
	*Candida albicans* planktonic	710 µM	[78]
	*Escherichia coli*	>5.5 mM	[58]
	*Escherichia coli* #916	2.78 mM	[69]
	*Escherichia coli*	1.78 mM	[57]
	*Escherichia coli* NCIMB 12210	8.0 mM	[68]
	*Escherichia coli* O157:H7	1.94 mM	[70]
	*Fusarium oxysporum*	>5.5 mM	[73]
	*Fusarium verticilioides*	>5.5 mM	[73]
	*Lactobacillus hilgardii* IFI-CA 49	4.44 mM	[71]
	*Lactobacillus rhamnosus* #299	<1.39 mM	[69]
	*Listeria monocytogenes*	16.1 mM	[46]
	*Morganella morganni*	>5.5 mM	[58]
	*Neisseria gonorrhoeae*	>5.5 mM	[58]
	*Pasteurella multocida*	5.5 mM	[58]
	*Pediococcus pentosaceus* IFI-CA 85	3.89 mM	[71]
	*Penicillium brevicompactum*	>5.5 mM	[73]
	*Penicillium expansum*	>5.5 mM	[73]
	*Proteus mirabilis*	>5.5 mM	[58]
	*Pseudomonas syringae* NCIMB 649	4.0 mM	[68]
	*Saccharomyces cerevisiae* 019 391	>8.0 mM	[68]
	*Salmonella typhimurium* #450	2.78 mM	[69]
	*Salmonella typhimurium* NRRL E4463	1.94 mM	[70]
	*Schizosaccharomyces pombe* 039 917	>8.0 mM	[68]
	*Sporobolomyces roseus* 043 529	>8.0 mM	[68]
	*Staphylococcus aureus* 209	694 µM	[77]
	*Staphylococcus aureus* # 917	694 µM	[69]
	*Staphylococcus aureus* NCTC 10657	2.22 mM	[70]
	*Streptococcus pyogenes* 10535	694 µM	[77]
ferulic acid (**7**)	*Aspergillus flavus*	>5.15 mM	[73]
	*Aspergillus flavus* UBA294	161 µM	[65]
	*Aspergillus fumigatus*	>5.15 mM	[73]
	*Aspergillus niger*	>10 mM	[80]
	*Aspergillus niger* ATCC 11394	322 µM	[65]
	*Aspergillus terreus* INM 031783	>1.3 mM	[65]
	*Bacillus cereus* No-8	2.06 mM	[70]
	*Bacillus subtilis*	6.0 mM	[80]
	*Bacillus subtilis* NCIMB 8649	2.0 mM	[68]
	*Candida albicans*	>10 mM	[80]
	*Candida albicans* ATCC 10231	659 µM	[81]
	*Candida krusei* ATCC 6258	659 µM	[81]
	*Enterococcus faecalis* ATCC 29212	659 µM	[81]
	*Escherichia coli*	>5.15 mM	[58]
	*Escherichia coli* ATCC 25922	1.3 mM	[81]
	*Escherichia coli* O157:H7	2.32 mM	[70]
	*Escherichia coli* CECT 434	515 µM	[79]
	*Escherichia coli* IFO13275	>5.0 mM	[94]
	*Escherichia coli* NCIMB 12210	2.0 mM	[68]
	*Fusarium oxysporum*	>5.15 mM	[73]
	*Fusarium verticilioides*	>5.15 mM	[73]
	*Klebsiella pneumoniae* RSKK 574	1.3 mM	[81]
	*Listeria monocytogenes*	13.9 mM	[46]
	*Listeria monocytogenes* ATCC 15313	6.44 mM	[79]
	*Morganella morganni*	>5.15 mM	[58]
	*Neisseria gonorrhoeae*	5.15 mM	[58]
	*Pasteurella multocida*	5.15 mM	[58]
	*Pediococcus pentosaceus* IFI-CA 85	4.63 mM	[71]
	*Penicillium brevicompactum*	>5.15 mM	[73]
	*Penicillium expansum*	>5.15 mM	[73]
	*Proteus mirabilis*	>5.15 mM	[58]
	*Pseudomonas aeruginosa* ATCC 10145	515 µM	[79]
	*Pseudomonas syringae* NCIMB 649	2.0 mM	[68]
	*Saccharomyces cerevisiae*	6.0 mM	[80]
	*Saccharomyces cerevisiae* 019 391	4.0 mM	[68]
	*Salmonella enteriditis* IFO3133	>5.0 mM	[94]
	*Salmonella typhimurium* NRRL E4463	2.06 mM	[70]
	*Schizosaccharomyces pombe* 039 917	8.0 mM	[68]
	*Sporobolomyces roseus* 043 529	2.0 mM	[68]
	*Staphylococcus aureus*	6.0 mM	[80]
	*Staphylococcus aureus* 209	644 µM	[77]
	*Staphylococcus aureus* ATCC 29213	1.3 mM	[81]
	*Staphylococcus aureus* CECT 976	5.7 mM	[79]
	*Staphylococcus aureus* IFO 12732	2.0 mM	[94]
	*Staphylococcus aureus* NCTC 10657	3.09 mM	[70]
	*Streptococcus pyogenes* 10535	644 µM	[77]
sinapic acid (**8**)	*Bacillus subtilis* NCIMB 8649	2.0 mM	[68]
	*Bacillus subtilis* FAD 110	1.3 mM	[83]
	*Escherichia coli* NCIMB 12210	2.0 mM	[68]
	*Escherichia coli* IFO13275	2.2 mM	[94]
	*Escherichia coli* AW 1.7	3.1 mM	[83]
	*Listeria innocua* ATCC 330909	1.3 mM	[83]
	*Listeria monocytogenes* ATCC 7644	900 µM	[83]
	*Pseudomonas fluorescens* ATCC 13525	2.7 mM	[83]
	*Pseudomonas syringae* NCIMB 649	4.0 mM	[68]
	*Saccharomyces cerevisiae* 019 391	>8.0 mM	[68]
	*Salmonella enteriditis* IFO3133	2.0 mM	[94]
	*Schizosaccharomyces pombe* 039 917	>8.0 mM	[68]
	*Sporobolomyces roseus* 043 529	>8.0 mM	[68]
	*Staphylococcus aureus* 209	558 µM	[77]
	*Staphylococcus aureus* IFO 12732	1.8 mM	[94]
	*Staphylococcus aureus* ATCC 6538	1.3 mM	[83]
	*Streptococcus pyogenes* 10535	558 µM	[77]
4-methoxycinnamic acid (**9**)	*Aspergillus niger*	50.4 µM	[44]
	*Bacillus subtilis*	203 µM	[44]
	*Candida albicans*	50.4 µM	[44]
	*Escherichia coli*	164 µM	[44]
	*Escherichia coli*	281 µM	[84]
	*Micrococcus luteus*	449 µM	[84]
	*Salmonella enteriditis*	337 µM	[84]
	*Staphylococcus aureus*	337 µM	[84]
	*Staphylococcus aureus*	203 µM	[44]
3,4-methylenedioxy-cinnamic acid (**10**)	*Mycobacterium tuberculosis* H_37_Rv	312 µM	[60]
	*Mycobacterium tuberculosis* H_37_Rv	>520 µM	[85]
4-nitrocinnamic acid (**11**)	*Bacillus subtilis* IFO 3009	891 µM	[86]
	*Escherichia coli* IFO 3301	794 µM	[86]
3-nitrocinnamic acid (**12**)	*Aspergillus niger*	43.5 µM	[44]
	*Bacillus subtilis*	203 µM	[44]
	*Candida albicans*	43.5 µM	[44]
	*Escherichia coli*	252 µM	[44]
	*Staphylococcus aureus*	252 µM	[44]
4-aminocinnamic acid (**13**)	*Bacillus subtilis* IFO 3009	602 µM	[86]
	*Escherichia coli* IFO 3301	708 µM	[86]
4-chlorocinnamic acid (**14**)	*Bacillus subtilis* IFO 3009	708 µM	[86]
	*Escherichia coli* IFO 3301	708 µM	[86]
4-*O*-prenylcoumaric acid (**16**)	*Mycobacterium tuberculosis* H_37_Rv	86.1 µM	[60]
4-*O*-geranylcoumaric acid (**17**)	*Mycobacterium tuberculosis* H_37_Rv	66.8 µM	[60]
3-*O*-prenylcoumaric acid (**18**)	*Mycobacterium tuberculosis* H_37_Rv	172 µM	[60]
2-*O*-prenylcoumaric acid (**19**)	*Mycobacterium tuberculosis* H_37_Rv	258 µM	[60]
3-prenyl-4-coumaric acid (= drupanin) (**20**)	*Aspergillus fumigatus* ATCC 26934	>1.1 mM	[92]
	*Aspergillus flavus* ATCC 9170	>1.1 mM	[92]
	*Aspergillus niger* ATCC 9029	>1.1 mM	[92]
	*Candida albicans* ATCC 10231	1.1 mM	[92]
	*Candida tropicalis* CEREMIC 131	>1.1 mM	[92]
	*Cryptococcus neoformans* ATCC 32264	1.1 mM	[92]
	*Epidermophyton floccosum* C114	215 µM	[92]
	*Escherichia coli* ATCC 25922	>1.1 mM	[92]
	*Microsporum canis* C112	431 µM	[92]
	*Microsporum gypseum* C115	431 µM	[92]
	*Staphylococcus aureus* LMS	>1.1 mM	[92]
	Methicillin-resistant *Staphylococcus aureus*	>1.1 mM	[92]
	*Trichophyton mentagrophytes* ATCC 9972	431 µM	[92]
	*Trichophyton rubrum* C113	431 µM	[92]
4-acetyl-3-prenyl-4-coumaric acid (**21**)	*Aspergillus fumigatus* ATCC 26934	>912 µM	[92]
	*Aspergillus flavus* ATCC 9170	>912 µM	[92]
	*Aspergillus niger* ATCC 9029	>912 µM	[92]
	*Candida albicans* ATCC 10231	>912 µM	[92]
	*Candida tropicalis* CEREMIC 131	>912 µM	[92]
	*Cryptococcus neoformans* ATCC 32264	>912 µM	[92]
	*Epidermophyton floccosum* C114	364 µM	[92]
	*Escherichia coli* ATCC 25922	>912 µM	[92]
	*Microsporum canis* C112	>912 µM	[92]
	*Microsporum gypseum* C115	912 µM	[92]
	*Staphylococcus aureus* LMS	>912 µM	[92]
	Methicillin-resistant *Staphylococcus aureus*	>912 µM	[92]
	*Trichophyton mentagrophytes* ATCC 9972	456 µM	[92]
	*Trichophyton rubrum* C113	456 µM	[92]
3,5-diprenyl-4-coumaric acid (**22**)	*Aspergillus fumigatus* ATCC 26934	>833 µM	[92]
	*Aspergillus flavus* ATCC 9170	>833 µM	[92]
	*Aspergillus niger* ATCC 9029	>833 µM	[92]
	*Candida albicans* ATCC 10231	833 µM	[92]
	*Candida tropicalis* CEREMIC 131	>833 µM	[92]
	*Cryptococcus neoformans* ATCC 32264	>833 µM	[92]
	*Epidermophyton floccosum* C114	166 µM	[92]
	*Escherichia coli* ATCC 25922	>833 µM	[92]
	*Microsporum canis* C112	>833 µM	[92]
	*Microsporum gypseum* C115	>833 µM	[92]
	*Staphylococcus aureus* LMS	833 µM	[92]
	Methicillin-resistant *Staphylococcus aureus*	833 µM	[92]
	*Trichophyton mentagrophytes* ATCC 9972	>833 µM	[92]
	*Trichophyton rubrum* C113	416 µM	[92]
4-acetyl-3,5-diprenyl-4-coumaric acid (**23**)	*Aspergillus fumigatus* ATCC 26934	>731 µM	[92]
	*Aspergillus flavus* ATCC 9170	>731 µM	[92]
	*Aspergillus niger* ATCC 9029	>731 µM	[92]
	*Candida albicans* ATCC 10231	>731 µM	[92]
	*Candida tropicalis* CEREMIC 131	>731 µM	[92]
	*Cryptococcus neoformans* ATCC 32264	>731 µM	[92]
	*Epidermophyton floccosum* C114	292 µM	[92]
	*Escherichia coli* ATCC 25922	>731 µM	[92]
	*Microsporum canis* C112	>731 µM	[92]
	*Microsporum gypseum* C115	>731 µM	[92]
	*Staphylococcus aureus* LMS	>731 µM	[92]
	Methicillin-resistant *Staphylococcus aureus*	>731 µM	[92]
	*Trichophyton mentagrophytes* ATCC 9972	731 µM	[92]
	*Trichophyton rubrum* C113	365 µM	[92]
*O*-acetylferulic acid (**24**)	*Candida albicans* ATCC 10231	540 µM	[81]
	*Candida krusei* ATCC 6258	540 µM	[81]
	*Enterococcus faecalis* ATCC 29212	540 µM	[81]
	*Escherichia coli* ATCC 25922	1.1 mM	[81]
	*Klebsiella pneumoniae* RSKK 574	1.1 mM	[81]
	*Staphylococcus aureus* ATCC 29213	1.1 mM	[81]

The compound 3,5-diprenyl-4-coumaric acid (**22**) was comparatively more active growth inhibitor of *E. floccosum* C114 achieving an MIC values of 166 µM [92]. Its *O*-acetyl derivative (**23**) was again slightly less active than the non-acetylated compound (Table 1), suggesting that the free phenolic OH is essential for potent activity of this class of molecules. The 4-*O*-acetyl derivative of ferulic acid, namely 4-*O*-acetylferulic acid (**24**), prepared by synthesis, inhibited the growth of *C. albicans*, *Candida krusei* and *Enterococcus faecalis* at 540 µM, but was less active against *S. aureus*, *E. coli* and *Klebsiella pneumoniae* [81]. Its activity was found to be comparatively similar to the antimicrobial effect of ferulic acid.

## 3. Natural and Synthetic Cinnamic Esters and Amides

### 3.1. Esters

**Figure 4:**
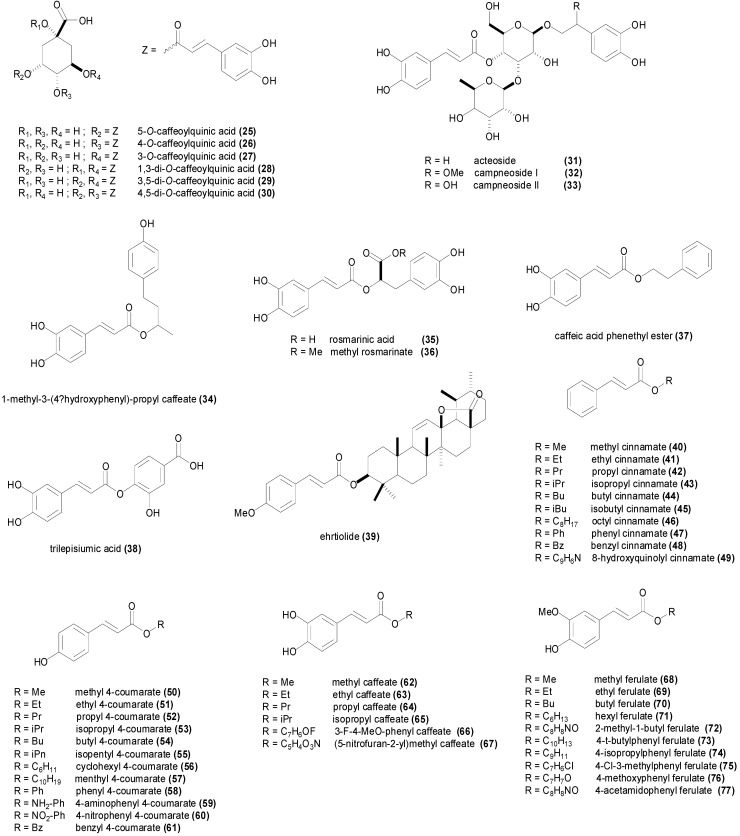
Chemical structures of cinnamic esters displaying antimicrobial activity.

The chlorogenic acids are a family of natural esters of hydroxycinnamic acids (coumaric, caffeic, ferulic and sinapic acids) with (−)-quinic acid [14]. The most common chlorogenic acid is 5-*O*-caffeoylquinic acid (**25**, Figure 4), which is abundant in coffee, black tea and mate but is also present in apples, pears and berries [95]. This chlorogenic acid isolated from artichoke, displayed MIC values of 564 µM against *B. subtilis*, *S. aureus*, *E. coli*, *S. typhimurium*, *P. aeruginosa* and *S. cerevisiae*, and lower MIC values of 282 µM against *Micrococcus luteus*, *Agrobacterium tumefaciens*, *Aspergillus niger*, *Penicillium oxalicum* and *Mucor mucedo* [96]. It was even more active against the fungi *Candida albicans*, *Candida lusitaniae*, *Saccharomyces carlsbergencis* and *Cladosporium cucumerinum* with an MIC value of 141 µM [96]. MIC values for the natural cinnamic esters are summarized in Table 2. However the effect of (**25**), isolated from roasted coffee beans, against *S. aureus* and *Streptococcus mutans* was much less marked in the study of Daglia *et al.*, with MIC values of 17.8 and 7.62 mM respectively [97]. The study of Xia *et al.*, isolated the three position isomers of caffeoylquinic acid from the *Prunus mume* seeds, and evaluated their antimicrobial activity against a panel of microorganisms, finding for (**25**) MIC values of 282 µM against *S. aureus*, 423 µM against *E. coli* and *C. albicans*, 564 µM against *S. enterica*, *S. cerevisiae* and *A. niger*, and an MIC value of 705 µM against *Vibrio parahaemolyticus* [98]. This natural product isolated from *Artemisia absinthium* inhibited completely the growth of *S. aureus* at a concentration of 361 µM, being even more active against *B. cereus*, *E. faecalis* with an MIC value 181 µM [99]. In addition 5-*O*-caffeoylquinic acid demonstrated biofilm formation inhibition in *S. aureus* and *E. faecalis*. Controversial results have also been reported for **25**, as the study of Alves *et al.* reported MIC values higher than 2.8 mM against all the evaluated microorganisms including *E. coli* [58]. The study of Lou *et al.*, published in 2011, showed MIC values ranging from 54 µM against *Streptococcus pneumoniae* and *Shigella disenteriae* to 226 µM against *E. coli* [100]. The methyl ester of **25**, namely methyl 5-*O*-caffeoylquinate isolated from the invasive plant *Ageratina adenophora*, inhibited the growth of *S. aureus*, *Bacillus thuringensis*, *E. coli* and *S. enterica* at a concentration of 89 µM [101], and was therefore comparatively more active than the parent chlorogenic acid. Transmission electron microscopy, membrane potential and nucleotide leakage studies on *Shigella disenteriae* led to the conclusion that the chlorogenic acid disrupted cell wall permeability and then depolarized the bacterial cell wall membrane causing cytoplasmic leakage [100].

4-*O*-Caffeoylquinic acid (**26**) (Figure 4 and Table 2) was found to be slightly less active than the 5-*O*-isomer with MIC values of 423 µM against both *S. aureus* and *E. coli*, 564 µM against both fungi *C. albicans* and *A. niger*, and 705 µM against *S. enterica* and *S. cerevisiae* [98]. In contrast with 5-*O*-caffeoylquinic acid, 3-*O*-caffeoylquinic acid (**27**) displayed more pronounced growth inhibitory activity against bacteria than fungi (Table 2), achieving MIC values of 282 µM against *E. coli* and *S. aureus* [98]. The literature reports of the antimicrobial activity of the 3-*O*- and 4-*O*-isomers are scarce, in contrast with the 5-*O*-isomer. The fact that the esters **25**, **26** and **27** have a different antimicrobial profile suggests that they have antimicrobial activity on their own, and the hydrolysis products caffeic and quinic acids, which could be released in the same amount, are not responsible for the antimicrobial activity of the three esters.

Among the chlorogenic acids formed by two caffeic acids residues, 1,3-di-*O*-caffeoylquinic acid (**28**), 3,5-di-*O*-caffeoylquinic acid (**29**) and 4,5-di-*O*-caffeoylquinic acid (**30**), the presence of caffeic residue in the position 3 of quinic acid increased the antifungal spectrum of activity, with **28** achieving MIC values below 200 µM against almost all fungi tested (Table 2), whereas the esterification in the position 5 of quinic acid increased the antibacterial potency of the acid, displaying MIC values of 194 µM *against E. coli* and *S. aureus* for **30** and even inhibiting completely the growth of *Micrococcus luteus* at 97 µM [96]. However discrepant MIC have also been reported. A report from 2011, published MIC values higher than 248 µM against *S. aureus*, *B. cereus* and higher than 496 µM against *E. coli* and *C. albicans* for **28**, **29** and **30** [99]. Moreover the study found an impressive synergism (FICI < 0.002) between **30** and several fluoroquinolone antibiotics against *S. aureus*. Four caffeic acid glycosides isolated from *Paulownia tomentosa* displayed antibacterial activity [102]. Campneoside I (**32**) demonstrated the highest activity against Gram-positive bacteria achieving MIC values around 200 µM against different species of *S. aureus*, while both acteoside (**31**) and campneoside II (**33**) showed less antistaphylococcal activity [102].

The caffeic acid ester **34** (Figure 4) isolated from *Zuccagnia punctata*, showed antifungal activity against the phytopathogenic fungi *Phomopsis longicolla* with an MIC value of only 19 µM, being less active against other fungi [103]. Another caffeic acid ester known as rosmarinic acid (**35**), and its methyl ester **36** showed growth inhibition of several species of bacteria with MIC values ranging between 801 µM and 6.94 mM [104]. In the study, the methyl ester **36** was slightly more active than the free acid **35**. However both rosmarinic acid (**35**) and its methyl ester **36** isolated from *Rabdosia serra*, were reported to have significantly higher MIC values against Gram-positive and Gram-negative bacteria [105], and surprisingly the methyl ester was found to be completely inactive with MIC values higher than 3.4 mM, whereas rosmarinic acid showed some activity [105] (Table 2). Moreover the study of Gohari *et al.*, showed that *Candida albicans* was more susceptible to rosmarinic acid with an MIC value of 694 µM, in comparison with *S. aureus*, *E. coli* and *Aspergillus niger* [106]. Caffeic acid phenethyl ester (**37**) is a biologically active component of propolis showing interesting anticancer activity [107]. This ester demonstrated antibacterial activity against *S. aureus*, *E. faecalis* and *L. monocytogenes* with MIC values ranging between 100 and 400 µM, but did not show growth inhibition against *P. aeruginosa* and *E. coli* up to a concentration of 800 µM [108]. Trilepisiumic acid (**38**) is an ester of caffeic acid and protocatechuic acid, isolated from *Trilepisium madagascariense*, that shows antifungal activity against *Candida albicans* ATCC 9002 (MIC = 202 µM) while being less active against other fungi and bacteria [109]. Two cinnamic esters were isolated from the root of *Ehretia longiflora*, and identified as ehretiolide (**39**) and arachidyl ferulate [110]. Ehretiolide (**39**) showed growth inhibitory activity against *M. tuberculosis* H_37_Rv (MIC = 41 µM), which is impressive for an unmodified natural product.

All the reported esters of cinnamic acid **40**–**49**, showed potent antifungal activity against *A. niger* and *C. albicans*, particularly isobutyl cinnamate (**45**) achieving MIC values of 12 and 14 µM respectively [44]. The MIC values of **40**–**49** against *A. niger* and *C. albicans* ranged between 12 and 61 µM, and between 14 and 61 µM respectively (Table 2). The MIC values of **40**–**49** were higher against Gram-positive and Gram-negative bacteria, ranging between 43 and 301 µM [44]. Isobutyl cinnamate (**45**), also showed the highest growth inhibitory activity against the bacterial species with MIC values between 43 and 50 µM. The esters of 4-coumaric acid **50**–**61** were comparatively less active compared to the esters of cinnamic acid, achieving MIC values between 67 µM and 2.3 mM against *A. niger*, and between 10 µM and 1.1 mM against *C. albicans* [111] (Table 2). The most active ester of 4-coumaric acid was 4-nitrophenyl 4-coumarate (**60**) showing MIC values of 46 µM against *B. subtilis*, *C. albicans*, *E. coli* and *S. aureus* [111]. The ester ethyl 4-methoxycinnamate, isolated from the plant *Kaempferia galanga*, showed anti-TB activity (MIC = 485 µM) against the virulent H_37_Rv and drug-resistant strains [112]. The esters of caffeic acid **62**–**67** were not assayed against different microorganisms, but only against some fungal species. Methyl caffeate (**62**) showed little antimicrobial activity against *Aspergillus* species with MIC values higher than 1.3 mM [65], however it was more active against *C. albicans* (MIC_50_ = 659 µM) [77,78].

**Table 2:** Minimum inhibitory concentration values of natural and synthetic cinnamic esters **25**–**77**.

Compound	Microorganism Strain	MIC	Refs.
5-*O*-caffeoylquinic acid (**25**)	*Agrobacterium tumefaciens* CGMCC 1.1415	282 µM	[96]
	*Aspergillus niger* ATCC 10553	564 µM	[98]
	*Aspergillus niger* CGMCC 3.316	282 µM	[96]
	*Bacillus subtilis* CGMCC 1.1849	564 µM	[96]
	*Bacillus subtilis* 9372	108 µM	[100]
	*Candida albicans* ATCC 10231	141 µM	[96]
	*Candida albicans* ATCC 14053	423 µM	[98]
	*Candida albicans* DAY185	181 µM	[99]
	*Candida lusitaniae* ATCC 2201	141 µM	[96]
	*Cladosporium cucumerinum* ATCC 11279	141 µM	[96]
	*Enterococcus faecalis* OGRF1	181 µM	[99]
	*Escherichia coli* ATCC 25922	216 µM	[100]
	*Escherichia coli* ATCC 25922	423 µM	[98]
	*Escherichia coli* CGMCC 1.90	564 µM	[96]
	*Micrococcus luteus* CGMCC 1.880	282 µM	[96]
	*Mucor mucedo* CGMCC 3.15	282 µM	[96]
	*Penicillium oxalicum* CGMCC 3.4022	282 µM	[96]
	*Pseudomonas aeruginosa* CG-MCC 1.2031	564 µM	[96]
	*Saccharomyces carlsbergensis* ATCC 2166	141 µM	[96]
	*Saccharomyces cerevisiae* ATCC 36858	564 µM	[98]
	*Saccharomyces cerevisiae* IFFI 1611	564 µM	[96]
	*Salmonella enterica* ATCC 13076	564 µM	[98]
	*Salmonella typhimurium* CGMCC 1.1190	564 µM	[96]
	*Salmonella typhimurium* 50013	108 µM	[100]
	*Shigella disenteriae* 51302	54 µM	[100]
	*Staphylococcus aureus*	17.8 mM	[97]
	*Staphylococcus aureus* 8325-4	361 µM	[99]
	*Staphylococcus aureus* 6538	108 µM	[100]
	*Staphylococcus aureus* ATCC 25923	282 µM	[98]
	*Staphylococcus aureus* ATCC 6358P	564 µM	[96]
	*Streptococcus mutans*	7.62 mM	[97]
	*Streptococcus pneumoniae* ATCC 49619	54 µM	[100]
	*Vibrio parahaemolyticus* ATCC 17802	705 µM	[98]
4-*O*-caffeoylquinic acid (**26**)	*Aspergillus niger* ATCC 10553	564 µM	[98]
	*Candida albicans* ATCC 14053	564 µM	[98]
	*Escherichia coli* ATCC 25922	423 µM	[98]
	*Saccharomyces cerevisiae* ATCC 36858	705 µM	[98]
	*Salmonella enterica* ATCC 13076	705 µM	[98]
	*Staphylococcus aureus* ATCC 25923	423 µM	[98]
3-*O*-caffeoylquinic acid (**27**)	*Aspergillus niger* ATCC 10553	423 µM	[98]
	*Candida albicans* ATCC 14053	423 µM	[98]
	*Escherichia coli* ATCC 25922	282 µM	[98]
	*Saccharomyces cerevisiae* ATCC 36858	564 µM	[98]
	*Salmonella enterica* ATCC 13076	564 µM	[98]
	*Staphylococcus aureus* ATCC 25923	282 µM	[98]
	*Vibrio parahaemolyticus* ATCC 17802	564 µM	[98]
1,3-di-*O*-caffeoylquinic acid (**28**)	*Agrobacterium tumefaciens* CGMCC 1.1415	194 µM	[96]
	*Aspergillus niger* CGMCC 3.316	194 µM	[96]
	*Bacillus subtilis* CGMCC 1.1849	387 µM	[96]
	*Candida albicans* ATCC 10231	194 µM	[96]
	*Candida lusitaniae* ATCC 2201	194 µM	[96]
	*Cladosporium cucumerinum* ATCC 11279	194 µM	[96]
	*Escherichia coli* CGMCC 1.90	194 µM	[96]
	*Micrococcus luteus* CGMCC 1.880	194 µM	[96]
	*Mucor mucedo* CGMCC 3.15	194 µM	[96]
	*Penicillium oxalicum* CGMCC 3.4022	194 µM	[96]
	*Pseudomonas aeruginosa* CG-MCC 1.2031	194 µM	[96]
	*Saccharomyces carlsbergensis* ATCC 2166	194 µM	[96]
	*Saccharomyces cerevisiae* IFFI 1611	387 µM	[96]
	*Salmonella typhimurium* CGMCC 1.1190	387 µM	[96]
	*Staphylococcus aureus* ATCC 6358P	387 µM	[96]
3,5-di-*O*-caffeoylquinic acid (**29**)	*Agrobacterium tumefaciens* CGMCC 1.1415	387 µM	[96]
	*Aspergillus niger* CGMCC 3.316	194 µM	[96]
	*Bacillus subtilis* CGMCC 1.1849	387 µM	[96]
	*Candida albicans* ATCC 10231	387 µM	[96]
	*Candida lusitaniae* ATCC 2201	387 µM	[96]
	*Cladosporium cucumerinum* ATCC 11279	194 µM	[96]
	*Escherichia coli* CGMCC 1.90	387 µM	[96]
	*Micrococcus luteus* CGMCC 1.880	194 µM	[96]
	*Mucor mucedo* CGMCC 3.15	194 µM	[96]
	*Penicillium oxalicum* CGMCC 3.4022	194 µM	[96]
	*Saccharomyces carlsbergensis* ATCC 2166	387 µM	[96]
	*Saccharomyces cerevisiae* IFFI 1611	387 µM	[96]
	*Salmonella typhimurium* CGMCC 1.1190	>387 µM	[96]
	*Staphylococcus aureus* ATCC 6358P	387 µM	[96]
4,5-di-*O*-caffeoylquinic acid (**30**)	*Agrobacterium tumefaciens* CGMCC 1.1415	387 µM	[96]
	*Aspergillus niger* CGMCC 3.316	387 µM	[96]
	*Bacillus subtilis* CGMCC 1.1849	387 µM	[96]
	*Candida lusitaniae* ATCC 2201	387 µM	[96]
	*Cladosporium cucumerinum* ATCC 11279	194 µM	[96]
	*Escherichia coli* CGMCC 1.90	194 µM	[96]
	*Micrococcus luteus* CGMCC 1.880	97 µM	[96]
	*Mucor mucedo* CGMCC 3.15	194 µM	[96]
	*Penicillium oxalicum* CGMCC 3.4022	194 µM	[96]
	*Saccharomyces carlsbergensis* ATCC 2166	387 µM	[96]
	*Saccharomyces cerevisiae* IFFI 1611	387 µM	[96]
	*Staphylococcus aureus* ATCC 6358P	194 µM	[96]
Acteoside (**31**)	*Enterobacter cloacae* P99	1.2 mM	[102]
	*Escherichia coli* 507E	4.8 mM	[102]
	*Klebsiella oxytosa* 1082E	1.2 mM	[102]
	*Klebsiella aerogenes* 1522E	2.4 mM	[102]
	*Pseudomonas aeruginosa* 9027	1.2 mM	[102]
	*Staphylococcus aureus*	3.2 mM	[102]
	*Staphylococcus aureus* SG511	600 µM	[102]
	*Streptococcus pyogenes* A308	1.2 mM	[102]
Campneoside I (**32**)	*Enterobacter cloacae* P99	>917 µM	[102]
	*Escherichia coli* 507E	>917 µM	[102]
	*Klebsiella oxytosa* 1082E	>917 µM	[102]
	*Klebsiella aerogenes* 1522E	>917 µM	[102]
	*Pseudomonas aeruginosa* 9027	>917 µM	[102]
	*Staphylococcus aureus*	200 µM	[102]
	*Staphylococcus aureus* SG511	229 µM	[102]
	*Streptococcus pyogenes* A308	229 µM	[102]
Campneoside II (**33**)	*Staphylococcus aureus*	2.0 mM	[102]
Caffeic acid ester (**34**)	*Phomopsis longicolla*	19 µM	[103]
Rosmarinic acid (**35**)	*Aspergillus niger* ATCC 16404	2.8 mM	[106]
	*Bacillus cereus* ATCC 10987	1.8 mM	[105]
	*Bacillus subtilis* ATCC 11060	1.8 mM	[105]
	*Candida albicans* ATCC 14053	694 µM	[106]
	*Corynebacterium* T25-17	6.9 mM	[104]
	*Enterococcus faecalis* C159-6	833 µM	[104]
	*Escherichia coli* ATCC 8739	1.4 mM	[106]
	*Listeria monocytogenes* ATCC 19115	1.8 mM	[105]
	*Mycobacterium smegmatis* 5003	3.3 mM	[104]
	*Pseudomonas aeruginosa* ATCC 27583	6.9 mM	[104]
	*Pseudomonas aeruginosa* ATCC 27853	1.8 mM	[105]
	*Staphylococcus aureus* ATCC 29213	888 µM	[105]
	*Staphylococcus aureus* ATCC 29737	1.4 mM	[106]
	*Staphylococcus epidermis* 5001	833 µM	[104]
	*Staphylococcus lugdunensis* T26A3	1.6 mM	[104]
	*Staphylococcus warneri* T12A12	3.3 mM	[104]
	*Stenotrophomonas maltophilia*	833 µM	[104]
Methyl rosmarinate (**36**)	*Bacillus cereus* ATCC 10987	>3.4 mM	[105]
	*Bacillus subtilis* ATCC 11060	>3.4 mM	[105]
	*Corynebacterium* T25-17	3.2 mM	[104]
	*Enterococcus faecalis* C159-6	801 µM	[104]
	*Listeria monocytogenes* ATCC 19115	>3.4 mM	[105]
	*Mycobacterium smegmatis* 5003	1.6 mM	[104]
	*Pseudomonas aeruginosa* ATCC 27583	3.2 mM	[104]
	*Pseudomonas aeruginosa* ATCC 27853	>3.4 mM	[105]
	*Staphylococcus aureus* ATCC 29213	>3.4 mM	[105]
	*Staphylococcus epidermis* 5001	801 µM	[104]
	*Staphylococcus lugdunensis* T26A3	1.6 mM	[104]
	*Staphylococcus warneri* T12A12	801 µM	[104]
	*Stenotrophomonas maltophilia*	801 µM	[104]
Caffeic acid phenethyl ester (**37**)	*Escherichia coli* ATCC 25922	>800 µM	[108]
	*Staphylococcus aureus* ATCC 6538P	100 µM	[108]
	*Enterococcus faecalis* ATCC 29212	400 µM	[108]
	*Listeria monocytogenes* ATCC 7644	400 µM	[108]
Methyl cinnamate (**40**)	*Aspergillus flavus* UBA 294	>1.5 mM	[65]
	*Aspergillus niger*	61 µM	[44]
	*Aspergillus niger* ATCC 11394	>1.5 mM	[65]
	*Aspergillus terreus* INM 031783	>1.5 mM	[65]
	*Bacillus subtilis*	301 µM	[44]
	*Candida albicans*	50 µM	[44]
	*Escherichia coli*	164 µM	[44]
	*Staphylococcus aureus*	252 µM	[44]
Ethyl cinnamate (**41**)	*Aspergillus niger*	61 µM	[44]
	*Bacillus subtilis*	203 µM	[44]
	*Candida albicans*	50 µM	[44]
	*Escherichia coli*	203 µM	[44]
	*Staphylococcus aureus*	252 µM	[44]
Propyl cinnamate (**42**)	*Aspergillus niger*	43 µM	[44]
	*Bacillus subtilis*	203 µM	[44]
	*Candida albicans*	59 µM	[44]
	*Escherichia coli*	203 µM	[44]
	*Staphylococcus aureus*	301 µM	[44]
Isopropyl cinnamate (**43**)	*Aspergillus niger*	43 µM	[44]
	*Bacillus subtilis*	164 µM	[44]
	*Candida albicans*	43 µM	[44]
	*Escherichia coli*	139 µM	[44]
	*Staphylococcus aureus*	139 µM	[44]
Butyl cinnamate (**44**)	*Aspergillus niger*	36 µM	[44]
	*Bacillus subtilis*	203 µM	[44]
	*Candida albicans*	61 µM	[44]
	*Escherichia coli*	203 µM	[44]
	*Staphylococcus aureus*	203 µM	[44]
Isobutyl cinnamate (**45**)	*Aspergillus niger*	12 µM	[44]
	*Bacillus subtilis*	43 µM	[44]
	*Candida albicans*	14 µM	[44]
	*Escherichia coli*	43 µM	[44]
	*Staphylococcus aureus*	50 µM	[44]
Octyl cinnamate (**46**)	*Aspergillus niger*	43 µM	[44]
	*Bacillus subtilis*	203 µM	[44]
	*Candida albicans*	43 µM	[44]
	*Escherichia coli*	164 µM	[44]
	*Staphylococcus aureus*	203 µM	[44]
Phenyl cinnamate (**47**)	*Aspergillus niger*	61 µM	[44]
	*Bacillus subtilis*	164 µM	[44]
	*Candida albicans*	43 µM	[44]
	*Escherichia coli*	252 µM	[44]
	*Staphylococcus aureus*	203 µM	[44]
Benzyl cinnamate (**48**)	*Aspergillus niger*	50 µM	[44]
	*Bacillus subtilis*	203 µM	[44]
	*Candida albicans*	43 µM	[44]
	*Escherichia coli*	203 µM	[44]
	*Staphylococcus aureus*	252 µM	[44]
8-Hydroxyquinolyl cinnamate (**49**)	*Aspergillus niger*	50 µM	[44]
	*Bacillus subtilis*	252 µM	[44]
	*Candida albicans*	61 µM	[44]
	*Escherichia coli*	164 µM	[44]
	*Staphylococcus aureus*	164 µM	[44]
Methyl 4-coumarate (**50**)	*Aspergillus flavus* UBA 294	702 µM	[65]
	*Aspergillus niger* ATCC 11394	702 µM	[65]
	*Aspergillus niger* MTCC 8189	247 µM	[111]
	*Aspergillus terreus* INM 031783	702 µM	[65]
	*Bacillus subtilis* MTCC 2063	247 µM	[111]
	*Candida albicans* MTCC 227	247 µM	[111]
	*Escherichia coli* MTCC 1652	247 µM	[111]
	*Staphylococcus aureus* MTCC 2901	247 µM	[111]
Ethyl 4-coumarate (**51**)	*Aspergillus niger* MTCC 8189	176 µM	[111]
	*Bacillus subtilis* MTCC 2063	176 µM	[111]
	*Candida albicans* MTCC 227	176 µM	[111]
	*Escherichia coli* MTCC 1652	176 µM	[111]
	*Staphylococcus aureus* MTCC 2901	176 µM	[111]
Propyl 4-coumarate (**52**)	*Aspergillus niger* MTCC 8189	2.3 mM	[111]
	*Bacillus subtilis* MTCC 2063	137 µM	[111]
	*Candida albicans* MTCC 227	137 µM	[111]
	*Escherichia coli* MTCC 1652	137 µM	[111]
	*Staphylococcus aureus* MTCC 2901	137 µM	[111]
Isopropyl 4-coumarate (**53**)	*Aspergillus niger* MTCC 8189	2.3 mM	[111]
	*Bacillus subtilis* MTCC 2063	137 µM	[111]
	*Candida albicans* MTCC 227	137 µM	[111]
	*Escherichia coli* MTCC 1652	2.3 mM	[111]
	*Staphylococcus aureus* MTCC 2901	137 µM	[111]
Butyl 4-coumarate (**54**)	*Aspergillus niger* MTCC 8189	1.9 mM	[111]
	*Bacillus subtilis* MTCC 2063	107 µM	[111]
	*Candida albicans* MTCC 227	107 µM	[111]
	*Escherichia coli* MTCC 1652	1.9 mM	[111]
	*Staphylococcus aureus* MTCC 2901	107 µM	[111]
Isopentyl 4-coumarate (**55**)	*Aspergillus niger* MTCC 8189	92 µM	[111]
	*Bacillus subtilis* MTCC 2063	92 µM	[111]
	*Candida albicans* MTCC 227	92 µM	[111]
	*Escherichia coli* MTCC 1652	1.4 mM	[111]
	*Staphylococcus aureus* MTCC 2901	92 µM	[111]
Cyclohexyl 4-coumarate (**56**)	*Aspergillus niger* MTCC 8189	78 µM	[111]
	*Bacillus subtilis* MTCC 2063	9.1 µM	[111]
	*Candida albicans* MTCC 227	1.1 mM	[111]
	*Escherichia coli* MTCC 1652	41 mM	[111]
	*Staphylococcus aureus* MTCC 2901	78 µM	[111]
Menthyl 4-coumarate (**57**)	*Aspergillus niger* MTCC 8189	1.9 mM	[111]
	*Bacillus subtilis* MTCC 2063	107 µM	[111]
	*Candida albicans* MTCC 227	107 µM	[111]
	*Escherichia coli* MTCC 1652	1.9 mM	[111]
	*Staphylococcus aureus* MTCC 2901	107 µM	[111]
Phenyl 4-coumarate (**58**)	*Aspergillus niger* MTCC 8189	1.2 mM	[111]
	*Bacillus subtilis* MTCC 2063	85 µM	[111]
	*Candida albicans* MTCC 227	10 µM	[111]
	*Escherichia coli* MTCC 1652	47 mM	[111]
	*Staphylococcus aureus* MTCC 2901	85 µM	[111]
4-aminophenyl 4-coumarate (**59**)	*Aspergillus niger* MTCC 8189	67 µM	[111]
	*Bacillus subtilis* MTCC 2063	8.5 µM	[111]
	*Candida albicans* MTCC 227	67 µM	[111]
	*Escherichia coli* MTCC 1652	905 µM	[111]
	*Staphylococcus aureus* MTCC 2901	67 µM	[111]
4-nitrophenyl 4-coumarate (**60**)	*Aspergillus niger* MTCC 8189	558 µM	[111]
	*Bacillus subtilis* MTCC 2063	46 µM	[111]
	*Candida albicans* MTCC 227	46 µM	[111]
	*Escherichia coli* MTCC 1652	46 µM	[111]
	*Staphylococcus aureus* MTCC 2901	46 µM	[111]
Benzyl 4-coumarate (**61**)	*Aspergillus niger* MTCC 8189	905 µM	[111]
	*Bacillus subtilis* MTCC 2063	67 µM	[111]
	*Candida albicans* MTCC 227	67 µM	[111]
	*Escherichia coli* MTCC 1652	905 µM	[111]
	*Staphylococcus aureus* MTCC 2901	67 µM	[111]
Methyl caffeate (**62**)	*Aspergillus flavus* UBA 294	>1.3 mM	[65]
	*Aspergillus niger* ATCC 11394	>1.3 mM	[65]
	*Aspergillus terreus* INM 031783	>1.3 mM	[65]
	*Candida albicans* ATCC 10231	MIC_50_ = 659 µM	[78]
Ethyl caffeate (**63**)	*Candida albicans* ATCC 10231	MIC_50_ = 615 µM	[78]
Propyl caffeate (**64**)	*Candida albicans* ATCC 10231	MIC_50_ = 576 µM	[78]
Isopropyl caffeate (**65**)	*Candida albicans* ATCC 10231	MIC_50_ = 576 µM	[78]
3-fluoro-4-methoxyphenyl caffeate (**66**)	*Candida albicans* ATCC 10231	MIC_50_ = 421 µM	[78]
(5-nitrofuran-2-yl)methyl caffeate (**67**)	*Candida albicans* ATCC 10231	MIC_50_ = 52 µM	[78]
Methyl ferulate (**68**)	*Aspergillus flavus* UBA 294	>1.2 mM	[65]
	*Aspergillus niger*	4.0 mM	[80]
	*Aspergillus niger* ATCC 11394	>1.2 mM	[65]
	*Aspergillus terreus* INM 031783	>1.2 mM	[65]
	*Bacillus subtilis*	6.0 mM	[80]
	*Candida albicans*	4.0 mM	[80]
	*Saccharomyces cerevisiae*	4.0 mM	[80]
	*Staphylococcus aureus*	6.0 mM	[80]
Ethyl ferulate (**69**)	*Aspergillus niger*	4.0 mM	[80]
	*Bacillus subtilis*	2.0 mM	[80]
	*Candida albicans*	4.0 mM	[80]
	*Saccharomyces cerevisiae*	4.0 mM	[80]
	*Staphylococcus aureus*	4.0 mM	[80]
Butyl ferulate (**70**)	*Aspergillus niger*	>10 mM	[80]
	*Bacillus subtilis*	500 µM	[80]
	*Candida albicans*	10 mM	[80]
	*Saccharomyces cerevisiae*	500 µM	[80]
	*Staphylococcus aureus*	500 µM	[80]
Hexyl ferulate (**71**)	*Aspergillus niger*	>10 mM	[80]
	*Bacillus subtilis*	63 µM	[80]
	*Candida albicans*	>10 mM	[80]
	*Saccharomyces cerevisiae*	>10 mM	[80]
	*Staphylococcus aureus*	125 µM	[80]
2-methyl-1-butyl ferulate (**72**)	*Aspergillus niger*	>10 mM	[80]
	*Bacillus subtilis*	125 µM	[80]
	*Candida albicans*	>10 mM	[80]
	*Saccharomyces cerevisiae*	250 µM	[80]
	*Staphylococcus aureus*	125 µM	[80]
4-*t*-butylphenyl ferulate (**73**)	*Candida albicans* ATCC 10231	391 µM	[81]
	*Candida krusei* ATCC 6258	391 µM	[81]
	*Enterococcus faecalis* ATCC 29212	391 µM	[81]
	*Escherichia coli* ATCC 25922	782 µM	[81]
	*Klebsiella pneumoniae* RSKK 574	782 µM	[81]
	*Staphylococcus aureus* ATCC 29213	782 µM	[81]
4-isopropylphenyl ferulate (**74**)	*Candida albicans* ATCC 10231	204 µM	[81]
	*Candida krusei* ATCC 6258	204 µM	[81]
	*Enterococcus faecalis* ATCC 29212	51 µM	[81]
	*Escherichia coli* ATCC 25922	818 µM	[81]
	*Klebsiella pneumoniae* RSKK 574	409 µM	[81]
	*Staphylococcus aureus* ATCC 29213	409 µM	[81]
4-chloro-3-methylphenyl ferulate (**75**)	*Candida albicans* ATCC 10231	201 µM	[81]
	*Candida krusei* ATCC 6258	201 µM	[81]
	*Enterococcus faecalis* ATCC 29212	50 µM	[81]
	*Escherichia coli* ATCC 25922	812 µM	[81]
	*Klebsiella pneumoniae* RSKK 574	401 µM	[81]
	*Staphylococcus aureus* ATCC 29213	<25 µM	[81]
4-methoxyphenyl ferulate (**76**)	*Candida albicans* ATCC 10231	425 µM	[81]
	*Candida krusei* ATCC 6258	425 µM	[81]
	*Enterococcus faecalis* ATCC 29212	850 µM	[81]
	*Escherichia coli* ATCC 25922	850 µM	[81]
	*Klebsiella pneumoniae* RSKK 574	850 µM	[81]
	*Staphylococcus aureus* ATCC 29213	850 µM	[81]
4-acetamidophenyl ferulate (**77**)	*Candida albicans* ATCC 10231	390 µM	[81]
	*Candida krusei* ATCC 6258	390 µM	[81]
	*Enterococcus faecalis* ATCC 29212	390 µM	[81]
	*Escherichia coli* ATCC 25922	780 µM	[81]
	*Klebsiella pneumoniae* RSKK 574	780 µM	[81]
	*Staphylococcus aureus* ATCC 29213	780 µM	[81]

The esters of caffeic acid showed moderate activity against *C. albicans*, and the most active was (5-nitrofuran-2-yl)methyl caffeate (**67**) with an MIC_50_ value of 52 µM against *C. albicans* [77,78]. The esters of ferulic acid **68**–**77** have been evaluated against different bacteria and fungi, and comparatively they have lower growth inhibitory activity compared to cinnamate and 4-coumarate esters. The MIC values of the ferulate esters **68**–**77** ranged between 201 µM and >10 mM against *C. albicans*, and between <25 µM and 6.0 mM against *S. aureus* [80,81]. The most active ferulate ester was identified to be 4-chloro-3-methylphenyl ferulate (**75**) achieving MIC values of 201 µM against *C. albicans* and *C. krusei*, of 50 µM against *E. faecalis*, 812 µM against *E. coli*, 401 µM against *K. pneumoniae*, and <25 µM against *S. aureus* [81].

### 3.2. Amides

**Figure 5:**
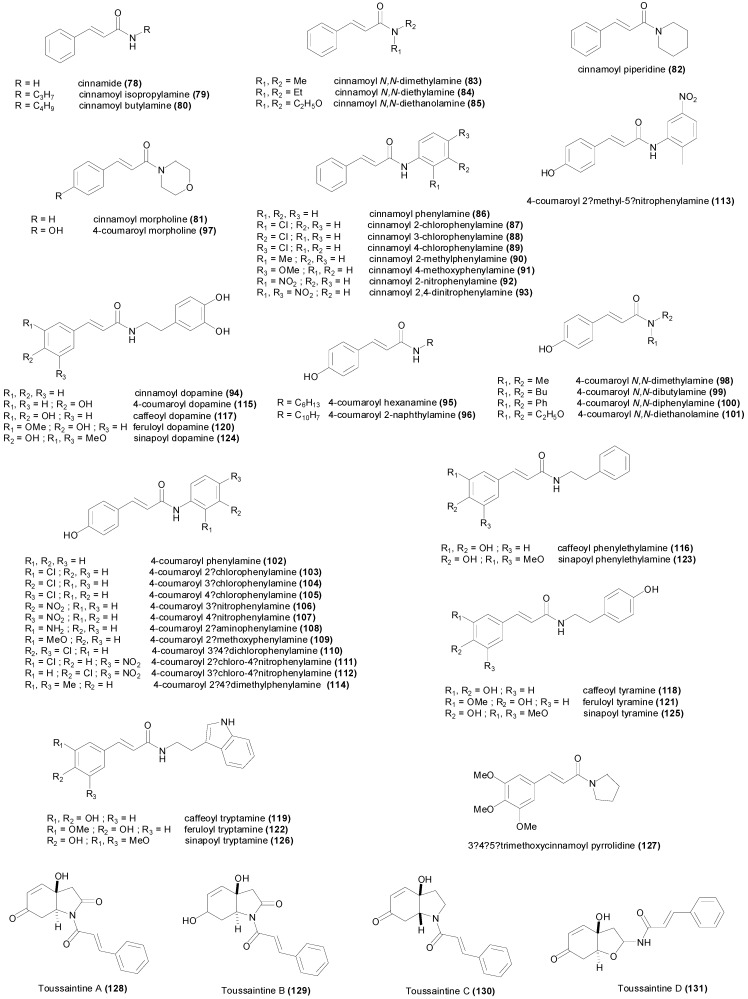
Chemical structures of the cinnamic amides **78**–**131** with antimicrobial activity

The simplest cinnamic amide is cinnamide (**78**) (Figure 5 and Table 3) which displayed growth inhibition against both fungi *A. niger* and *C. albicans* at a concentration of 60.8 µM, while being less active against bacteria, showing an MIC value of 252 µM against *B. subtilis*, *E. coli* and *S. aureus* [44]. All the cinnamoyl amides **79**–**94** were found to have a more potent effect against fungi compared to bacteria, except cinnamoyl dopamine (**94**) which showed little antimicrobial activity (MIC = 1.76 mM) [77]. Among the screened cinnamoyl amides, the compound with the highest antifungal activity was identified to be cinnamoyl *N*,*N*-diethylamide (**84**) achieving MIC values of 14.3 and 36.3 µM against *A. niger* and *C. albicans* respectively [44]. The most potent antibacterial cinnamoyl amide was cinnamoyl 2-methylphenylamine (**90**) with MIC values of 114, 139 and 139 µM against *B. subtilis*, *E. coli* and *S. aureus* respectively.

The 4-coumaroyl amides **95**–**115** were tested for antimicrobial activity, and the MIC results suggest that they are comparatively more active inhibitors of microbial growth than the cinnamoyl amides (Table 3). The 4-coumaroyl amides do not show a higher specificity for fungi compared to bacteria as was observed for the cinnamoyl amides. The MIC values of the 4-coumaroyl amides **95**–**115** ranged between 929 nM and 3.6 mM against bacteria, and between 6.3 µM and 18 mM against fungal species [77,111]. The 4-coumaroyl amide displaying the lowest MIC value against bacteria, was found to be 4-coumaroyl 2'-chloro-4'-nitrophenylamine (**111**) achieving an MIC value of 929 nM against *B. subtilis* MTCC 2063 [111], while the lowest MIC value against fungi was 6.3 µM attained by 4-coumaroyl 2'-nitrophenylamine (**103**) against *C. albicans* MTCC 227 [111]. Most of the 4-coumaroyl amides displayed higher activity against *B. subtilis* and *S. aureus* bacteria compared to *E. coli* (Table 3).

The caffeoyl amides **116**–**119** (Figure 5) showed moderate antimicrobial activity (Table 3) with MIC values ranging between 194 µM and 1.76 mM [77]. The lowest MIC value (194 µM) was reported for caffeoyl tryptamine (**119**) against *S. pyogenes* 10535, however the MIC values for the same amide against other bacteria were comparatively higher, being 1.55 mM against *B. subtilis* 1A95, *C. albicans* 62 and *L. monocytogenes* C12 [77]. The amides caffeoyl dopamine (**117**) and caffeoyl tyramine (**118**) showed growth inhibition against Gram-positive, Gram-negative and fungal species with MIC values between 396 and 793 µM, and 418 and 836 µM respectively, whereas the other caffeoyl amides displayed MIC values higher than 1.0 mM against at least one microorganism (Table 3). The three evaluated feruloyl amides **120**–**122** showed a very similar pattern of antimicrobial activity [77]. The feruloyl amides showed to be growth inhibitors of *S. aureus* 209 with MIC values between 190 and 372 µM. They were equally active against *S. pyogenes* but much less against *B. subtilis* (MIC around 743–798 µM), and *C. albicans* and *L. monocytogenes*. Interestingly all the sinapoyl amides **123**–**126** showed selective growth inhibition of Gram-positive cocci bacteria (MIC between 171 and 696 µM), while being much less effective inhibitors of *B. subtilis*, *L. monocytogenes* or *C. albicans* (MIC between 1.36 and 1.53 mM) [77].

The *Piper* amides, which are one of the major phytochemicals present in the medicinally important *Piper* species displayed a broad variety of biological activities [113]. The carboxylic acid residues of some *Piper* amides are in fact substituted cinnamic acids.

Piplartine (Figure 1), originally isolated from the roots of *Piper tuberculatum* [18], demonstrated antibacterial activity against *K. pneumoniae*, *P. aeruginosa* and *S. aureus*, however no MIC values were reported [114]. Among the amides isolated from the seeds of *Piper tuberculatum*, piplartine showed antifungal activity against *Cladosporium sphaerospermun* but no MIC value was determined [115,116]. The amide 3',4',5'-trimethoxycinnamoyl pyrrolidine (**127**), which is related to piplartine, was isolated from *Piper sarmentosum*, and showed MIC values higher than 600 µM against *M. tuberculosis* H_37_Ra [117,118]. The toussaintines are amides of cinnamic acid with indolidinones or benzofuranones, obtained from the African plant *Toussaintia orientalis* [119]. The toussaintine B (**129**) inhibited both *S. aureus* and *E. coli* with a low MIC value of 67 µM, while toussaintine A (**128**) inhibited *E. coli* at 34 µM but did not inhibit *S. aureus*, and toussaintine D (**131**) inhibited *S. aureus* at 17 µM but did not inhibit *E. coli* [119]. Reports of natural cinnamic amides displaying antimicrobial activity were very scarce in the literature.

**Table 3:** Minimum inhibitory concentration values of cinnamic amides **78**–**131**.

Compound	Microorganism Strain	MIC	Refs.
Cinnamide (**78**)	*Aspergillus niger*	60.8 µM	[44]
	*Bacillus subtilis*	252 µM	[44]
	*Candida albicans*	60.8 µM	[44]
	*Escherichia coli*	252 µM	[44]
	*Staphylococcus aureus*	252 µM	[44]
Cinnamoyl isopropylamine (**79**)	*Aspergillus niger*	60.8 µM	[44]
	*Bacillus subtilis*	164 µM	[44]
	*Candida albicans*	50.4 µM	[44]
	*Escherichia coli*	164 µM	[44]
	*Staphylococcus aureus*	252 µM	[44]
Cinnamoyl butylamine (**80**)	*Aspergillus niger*	43.5 µM	[44]
	*Bacillus subtilis*	203 µM	[44]
	*Candida albicans*	43.5 µM	[44]
	*Escherichia coli*	203 µM	[44]
	*Staphylococcus aureus*	164 µM	[44]
Cinnamoyl morpholine (**81**)	*Aspergillus niger*	60.8 µM	[44]
	*Bacillus subtilis*	164 µM	[44]
	*Candida albicans*	60.8 µM	[44]
	*Escherichia coli*	203 µM	[44]
	*Staphylococcus aureus*	203 µM	[44]
Cinnamoyl piperidine (**82**)	*Aspergillus niger*	43.5 µM	[44]
	*Bacillus subtilis*	164 µM	[44]
	*Candida albicans*	50.4 µM	[44]
	*Escherichia coli*	164 µM	[44]
	*Staphylococcus aureus*	139 µM	[44]
Cinnamoyl *N*,*N*-dimethylamine (**83**)	*Aspergillus niger*	50.4 µM	[44]
	*Bacillus subtilis*	139 µM	[44]
	*Candida albicans*	43.5 µM	[44]
	*Escherichia coli*	139 µM	[44]
	*Staphylococcus aureus*	139 µM	[44]
Cinnamoyl *N*,*N*-diethylamine (**84**)	*Aspergillus niger*	14.3 µM	[44]
	*Bacillus subtilis*	203 µM	[44]
	*Candida albicans*	36.3 µM	[44]
	*Escherichia coli*	203 µM	[44]
	*Staphylococcus aureus*	252 µM	[44]
Cinnamoyl *N*,*N*-diethanolamine (**85**)	*Aspergillus niger*	60.8 µM	[44]
	*Bacillus subtilis*	301 µM	[44]
	*Candida albicans*	86 µM	[44]
	*Escherichia coli*	301 µM	[44]
	*Staphylococcus aureus*	301 µM	[44]
Cinnamoyl phenylamine (**86**)	*Aspergillus niger*	73.6 µM	[44]
	*Bacillus subtilis*	301 µM	[44]
	*Candida albicans*	86 µM	[44]
	*Escherichia coli*	301 µM	[44]
	*Staphylococcus aureus*	203 µM	[44]
Cinnamoyl 2-chlorophenylamine (**87**)	*Aspergillus niger*	50.4 µM	[44]
	*Bacillus subtilis*	301 µM	[44]
	*Candida albicans*	60.8 µM	[44]
	*Escherichia coli*	301 µM	[44]
	*Staphylococcus aureus*	301 µM	[44]
Cinnamoyl 3-chlorophenylamine (**88**)	*Aspergillus niger*	43.5 µM	[44]
	*Bacillus subtilis*	203 µM	[44]
	*Candida albicans*	60.8 µM	[44]
	*Escherichia coli*	164 µM	[44]
	*Staphylococcus aureus*	203 µM	[44]
Cinnamoyl 4-chlorophenylamine (**89**)	*Aspergillus niger*	50.4 µM	[44]
	*Bacillus subtilis*	301 µM	[44]
	*Candida albicans*	60.8 µM	[44]
	*Escherichia coli*	301 µM	[44]
	*Staphylococcus aureus*	301 µM	[44]
Cinnamoyl 2-methylphenylamine (**90**)	*Aspergillus niger*	73.6 µM	[44]
	*Bacillus subtilis*	114 µM	[44]
	*Candida albicans*	86 µM	[44]
	*Escherichia coli*	139 µM	[44]
	*Staphylococcus aureus*	139 µM	[44]
Cinnamoyl 4-methoxyphenylamine (**91**)	*Aspergillus niger*	50.4 µM	[44]
	*Bacillus subtilis*	203 µM	[44]
	*Candida albicans*	50.4 µM	[44]
	*Escherichia coli*	203 µM	[44]
	*Staphylococcus aureus*	164 µM	[44]
Cinnamoyl 2-nitrophenylamine (**92**)	*Aspergillus niger*	86 µM	[44]
	*Bacillus subtilis*	164 µM	[44]
	*Candida albicans*	73.6 µM	[44]
	*Escherichia coli*	203 µM	[44]
	*Staphylococcus aureus*	139 µM	[44]
Cinnamoyl 2,4-dinitrophenylamine (**93**)	*Aspergillus niger*	50.4 µM	[44]
	*Bacillus subtilis*	164 µM	[44]
	*Candida albicans*	86 µM	[44]
	*Escherichia coli*	164 µM	[44]
	*Staphylococcus aureus*	164 µM	[44]
Cinnamoyl dopamine (**94**)	*Bacillus subtilis* 1A95	1.76 mM	[77]
	*Candida albicans* 62	1.76 mM	[77]
	*Listeria monocytogenes* C12	1.76 mM	[77]
	*Staphylococcus aureus* 209	1.76 mM	[77]
	*Streptococcus pyogenes* 10535	441 µM	[77]
4-coumaroyl hexanamine (**95**)	*Aspergillus niger* MTCC 8189	72.5 µM	[111]
	*Bacillus subtilis* MTCC 2063	9.1 µM	[111]
	*Candida albicans* MTCC 227	72.5 µM	[111]
	*Escherichia coli* MTCC 1652	72.5 µM	[111]
	*Staphylococcus aureus* MTCC 2901	72.5 µM	[111]
4-coumaroyl 2-naphtylamine (**96**)	*Aspergillus niger* MTCC 8189	558 µM	[111]
	*Bacillus subtilis* MTCC 2063	46.2 µM	[111]
	*Candida albicans* MTCC 227	15.5 mM	[111]
	*Escherichia coli* MTCC 1652	46.2 µM	[111]
	*Staphylococcus aureus* MTCC 2901	5.9 µM	[111]
4-coumaroyl morpholine (**97**)	*Aspergillus niger* MTCC 8189	1.4 mM	[111]
	*Bacillus subtilis* MTCC 2063	91.6 µM	[111]
	*Candida albicans* MTCC 227	91.6 µM	[111]
	*Escherichia coli* MTCC 1652	91.6 µM	[111]
	*Staphylococcus aureus* MTCC 2901	1.4 mM	[111]
4-coumaroyl *N*,*N*-dimethylamine (**98**)	*Aspergillus niger* MTCC 8189	191 µM	[111]
	*Bacillus subtilis* MTCC 2063	191 µM	[111]
	*Candida albicans* MTCC 227	191 µM	[111]
	*Escherichia coli* MTCC 1652	3.6 mM	[111]
	*Staphylococcus aureus* MTCC 2901	3.6 mM	[111]
4-coumaroyl *N*,*N*-dibutylamine (**99**)	*Aspergillus niger* MTCC 8189	676 µM	[111]
	*Bacillus subtilis* MTCC 2063	53.6 µM	[111]
	*Candida albicans* MTCC 227	53.6 µM	[111]
	*Escherichia coli* MTCC 1652	676 µM	[111]
	*Staphylococcus aureus* MTCC 2901	53.6 µM	[111]
4-coumaroyl *N*,*N*-diphenylamine (**100**)	*Aspergillus niger* MTCC 8189	386 µM	[111]
	*Bacillus subtilis* MTCC 2063	5.0 µM	[111]
	*Candida albicans* MTCC 227	34.6 µM	[111]
	*Escherichia coli* MTCC 1652	5.0 µM	[111]
	*Staphylococcus aureus* MTCC 2901	34.6 µM	[111]
4-coumaroyl *N*,*N*-diethanolamine (**101**)	*Aspergillus niger* MTCC 8189	72.5 µM	[111]
	*Bacillus subtilis* MTCC 2063	9.1 µM	[111]
	*Candida albicans* MTCC 227	72.5 µM	[111]
	*Escherichia coli* MTCC 1652	72.5 µM	[111]
	*Staphylococcus aureus* MTCC 2901	72.5 µM	[111]
4-coumaroyl phenylamine (**102**)	*Aspergillus niger* MTCC 8189	1.2 mM	[111]
	*Bacillus subtilis* MTCC 2063	84.7 µM	[111]
	*Candida albicans* MTCC 227	84.7 µM	[111]
	*Escherichia coli* MTCC 1652	84.7 µM	[111]
	*Staphylococcus aureus* MTCC 2901	10.3 µM	[111]
4-coumaroyl 2'-nitrophenylamine (**103**)	*Aspergillus niger* MTCC 8189	46.2 µM	[111]
	*Bacillus subtilis* MTCC 2063	46.2 µM	[111]
	*Candida albicans* MTCC 227	6.3 µM	[111]
	*Escherichia coli* MTCC 1652	46.2 µM	[111]
	*Staphylococcus aureus* MTCC 2901	46.2 µM	[111]
4-coumaroyl 3'-chlorophenylamine (**104**)	*Aspergillus niger* MTCC 8189	676 µM	[111]
	*Bacillus subtilis* MTCC 2063	7.1 µM	[111]
	*Candida albicans* MTCC 227	53.6 µM	[111]
	*Escherichia coli* MTCC 1652	53.6 µM	[111]
	*Staphylococcus aureus* MTCC 2901	7.1 µM	[111]
4-coumaroyl 4'-chlorophenylamine (**105**)	*Aspergillus niger* MTCC 8189	676 µM	[111]
	*Bacillus subtilis* MTCC 2063	7.1 µM	[111]
	*Candida albicans* MTCC 227	53.6 µM	[111]
	*Escherichia coli* MTCC 1652	53.6 µM	[111]
	*Staphylococcus aureus* MTCC 2901	53.6 µM	[111]
4-coumaroyl 3'-nitrophenylamine (**106**)	*Aspergillus niger* MTCC 8189	46.2 µM	[111]
	*Bacillus subtilis* MTCC 2063	46.2 µM	[111]
	*Candida albicans* MTCC 227	46.2 µM	[111]
	*Escherichia coli* MTCC 1652	558 µM	[111]
	*Staphylococcus aureus* MTCC 2901	46.2 µM	[111]
4-coumaroyl 4'-nitrophenylamine (**107**)	*Aspergillus niger* MTCC 8189	18 mM	[111]
	*Bacillus subtilis* MTCC 2063	46.2 µM	[111]
	*Candida albicans* MTCC 227	46.2 µM	[111]
	*Escherichia coli* MTCC 1652	46.2 µM	[111]
	*Staphylococcus aureus* MTCC 2901	46.2 µM	[111]
4-coumaroyl 2'-aminophenylamine (**108**)	*Aspergillus niger* MTCC 8189	905 µM	[111]
	*Bacillus subtilis* MTCC 2063	67.2 µM	[111]
	*Candida albicans* MTCC 227	67.2 µM	[111]
	*Escherichia coli* MTCC 1652	67.2 µM	[111]
	*Staphylococcus aureus* MTCC 2901	67.2 µM	[111]
4-coumaroyl 2'-methoxyphenylamine (**109**)	*Aspergillus niger* MTCC 8189	744 µM	[111]
	*Bacillus subtilis* MTCC 2063	7.5 µM	[111]
	*Candida albicans* MTCC 227	57.7 µM	[111]
	*Escherichia coli* MTCC 1652	57.7 µM	[111]
	*Staphylococcus aureus* MTCC 2901	57.7 µM	[111]
4-coumaroyl 3',4'-dichlorophenylamine (**110**)	*Aspergillus niger* MTCC 8189	422 µM	[111]
	*Bacillus subtilis* MTCC 2063	37.1 µM	[111]
	*Candida albicans* MTCC 227	37.1 µM	[111]
	*Escherichia coli* MTCC 1652	37.1 µM	[111]
	*Staphylococcus aureus* MTCC 2901	37.1 µM	[111]
4-coumaroyl 2'-chloro-4'-nitrophenylamine (**111**)	*Aspergillus niger* MTCC 8189	352 µM	[111]
	*Bacillus subtilis* MTCC 2063	929 nM	[111]
	*Candida albicans* MTCC 227	32.2 µM	[111]
	*Escherichia coli* MTCC 1652	32.2 µM	[111]
	*Staphylococcus aureus* MTCC 2901	352 µM	[111]
4-coumaroyl 3'-chloro-4'-nitrophenylamine (**112**)	*Aspergillus niger* MTCC 8189	32.2 µM	[111]
	*Bacillus subtilis* MTCC 2063	4.7 µM	[111]
	*Candida albicans* MTCC 227	32.2 µM	[111]
	*Escherichia coli* MTCC 1652	4.7 µM	[111]
	*Staphylococcus aureus* MTCC 2901	32.2 µM	[111]
4-coumaroyl 2'-methyl-5'-nitrophenylamine (**113**)	*Aspergillus niger* MTCC 8189	39.9 µM	[111]
	*Bacillus subtilis* MTCC 2063	39.9 µM	[111]
	*Candida albicans* MTCC 227	39.9 µM	[111]
	*Escherichia coli* MTCC 1652	463 µM	[111]
	*Staphylococcus aureus* MTCC 2901	39.9 µM	[111]
4-coumaroyl 2',4'-dimethylphenylamine (**114**)	*Aspergillus niger* MTCC 8189	744 µM	[111]
	*Bacillus subtilis* MTCC 2063	7.55 µM	[111]
	*Candida albicans* MTCC 227	57.7 µM	[111]
	*Escherichia coli* MTCC 1652	744 µM	[111]
	*Staphylococcus aureus* MTCC 2901	57.7 µM	[111]
4-coumaroyl dopamine (**115**)	*Bacillus subtilis* 1A95	1.67 mM	[77]
	*Candida albicans* 62	1.67 mM	[77]
	*Listeria monocytogenes* C12	1.67 mM	[77]
	*Staphylococcus aureus* 209	418 µM	[77]
	*Streptococcus pyogenes* 10535	418 µM	[77]
Caffeoyl phenylethylamine (**116**)	*Bacillus subtilis* 1A95	1.76 mM	[77]
	*Candida albicans* 62	882 µM	[77]
	*Listeria monocytogenes* C12	441 µM	[77]
	*Staphylococcus aureus* 209	882 µM	[77]
	*Streptococcus pyogenes* 10535	882 µM	[77]
Caffeoyl dopamine (**117**)	*Bacillus subtilis* 1A95	793 µM	[77]
	*Candida albicans* 62	396 µM	[77]
	*Listeria monocytogenes* C12	793 µM	[77]
	*Staphylococcus aureus* 209	793 µM	[77]
	*Streptococcus pyogenes* 10535	793 µM	[77]
Caffeoyl tyramine (**118**)	*Bacillus subtilis* 1A95	836 µM	[77]
	*Candida albicans* 62	418 µM	[77]
	*Listeria monocytogenes* C12	836 µM	[77]
	*Staphylococcus aureus* 209	836 µM	[77]
	*Streptococcus pyogenes* 10535	836 µM	[77]
Caffeoyl tryptamine (**119**)	*Bacillus subtilis* 1A95	1.55 mM	[77]
	*Candida albicans* 62	1.55 mM	[77]
	*Listeria monocytogenes* C12	1.55 mM	[77]
	*Staphylococcus aureus* 209	388 µM	[77]
	*Streptococcus pyogenes* 10535	194 µM	[77]
Feruloyl dopamine (**120**)	*Bacillus subtilis* 1A95	759 µM	[77]
	*Candida albicans* 62	1.52 mM	[77]
	*Listeria monocytogenes* C12	1.52 mM	[77]
	*Staphylococcus aureus* 209	190 µM	[77]
	*Streptococcus pyogenes* 10535	380 µM	[77]
Feruloyl tyramine (**121**)	*Bacillus subtilis* 1A95	798 µM	[77]
	*Candida albicans* 62	1.59 mM	[77]
	*Listeria monocytogenes* C12	1.59 mM	[77]
	*Staphylococcus aureus* 209	199 µM	[77]
	*Streptococcus pyogenes* 10535	399 µM	[77]
Feruloyl tryptamine (**122**)	*Bacillus subtilis* 1A95	743 µM	[77]
	*Candida albicans* 62	1.49 mM	[77]
	*Listeria monocytogenes* C12	1.49 mM	[77]
	*Staphylococcus aureus* 209	372 µM	[77]
	*Streptococcus pyogenes* 10535	372 µM	[77]
Sinapoyl phenylethylamine (**123**)	*Bacillus subtilis* 1A95	1.53 mM	[77]
	*Candida albicans* 62	1.53 mM	[77]
	*Listeria monocytogenes* C12	1.53 mM	[77]
	*Staphylococcus aureus* 209	382 µM	[77]
	*Streptococcus pyogenes* 10535	382 µM	[77]
Sinapoyl dopamine (**124**)	*Bacillus subtilis* 1A95	1.39 mM	[77]
	*Candida albicans* 62	1.39 mM	[77]
	*Listeria monocytogenes* C12	1.39 mM	[77]
	*Staphylococcus aureus* 209	696 µM	[77]
	*Streptococcus pyogenes* 10535	696 µM	[77]
Sinapoyl tyramine (**125**)	*Bacillus subtilis* 1A95	1.46 mM	[77]
	*Candida albicans* 62	1.46 mM	[77]
	*Listeria monocytogenes* C12	1.46 mM	[77]
	*Staphylococcus aureus* 209	182 µM	[77]
	*Streptococcus pyogenes* 10535	182 µM	[77]
Sinapoyl tryptamine (**126**)	*Bacillus subtilis* 1A95	1.36 mM	[77]
	*Candida albicans* 62	1.36 mM	[77]
	*Listeria monocytogenes* C12	1.36 mM	[77]
	*Staphylococcus aureus* 209	682 µM	[77]
	*Streptococcus pyogenes* 10535	171 µM	[77]
3',4',5'-trimethoxycinnamoyl pyrrolidine (**127**)	*Mycobacterium tuberculosis* H_37_Ra	600 µM	[118]
Toussaintine A (**128**)	*Escherichia coli* DSM 1103	34 µM	[119]
	*Staphylococcus aureus* ATCC 25923	>136 µM	[119]
Toussaintine B (**129**)	*Escherichia coli* DSM 1103	67 µM	[119]
	*Staphylococcus aureus* ATCC 25923	67 µM	[119]
Toussaintine C (**130**)	*Escherichia coli* DSM 1103	34 µM	[119]
	*Staphylococcus aureus* ATCC 25923	>136 µM	[119]
Toussaintine D (**131**)	*Escherichia coli* DSM 1103	>136 µM	[119]
	*Staphylococcus aureus* ATCC 25923	17 µM	[119]

## 4. Natural and Synthetic Cinnamic Aldehydes and Alcohols

**Figure 6:**
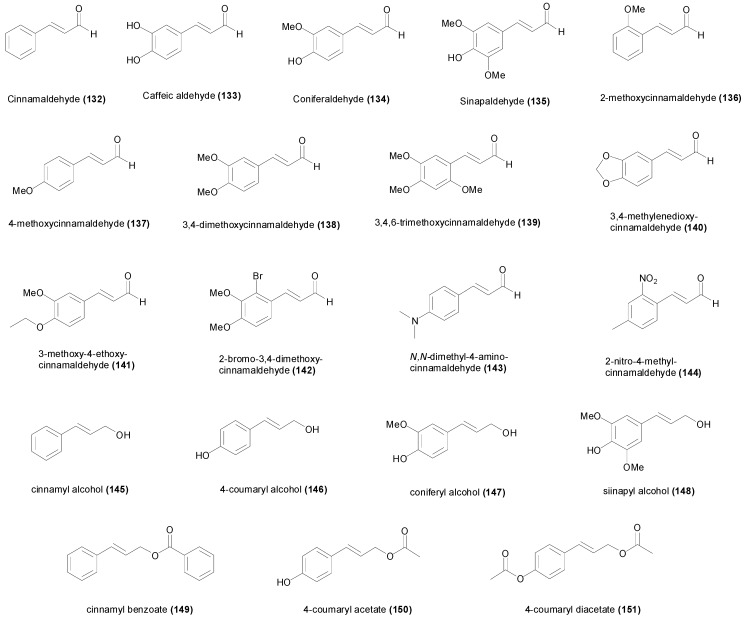
Chemical structures of the cinnamic aldehydes, alcohols and related natural products.

Cinnamaldehyde (**132**) (Figure 6, Table 4) which is abundantly obtained from the essential oils of cinnamon [12,120], has demonstrated a significant antimicrobial activity particularly against Gram-negative bacteria. The aldehyde was found to be very active against *Helicobacter pylori* and *E. coli* with MIC values of 15 and 7.6 µM respectively [121]. Similar MIC values of 10.6 µM and < 1.5 µM have been reported against *P. aeruginosa* and *E. coli* respectively [122]. However controversial results have appeared for this compound, as other reports published higher MIC values against *E. coli* [12,123,124,125]. Against the *Clostridium difficile* bacteria, the aldehyde showed complete growth inhibition at a concentration of 605 µM [13]. A similar MIC value was observed against *Legionella pneumophila*, the causative bacteria of Legionnaire’s disease [126]. Cinnamaldehyde has also found to be more active against *S. aureus* than Gram-negative bacteria [124], and interestingly the MIC values against a susceptible *S. aureus* and a methicillin-resistant *S. aureus* (MRSA) were exactly the same. The antibiotic clindamycin actually was found to be synergistic with cinnamaldehyde, with a FICI value of 0.3125 [124]. In addition, cinnamaldehyde showed interaction with the FtsZ cell division protein using isothermal titration calorimetry, and inhibited the formation of FtsZ assembly in a dose-dependent manner, which phenotypically translated into elongated cells [124]. A report from 2003, showed that cinnamic aldehyde prevented *Bacillus cereus* daughter cells separation during division, forming filamentous cells [127], a result correlating with inhibition of FtsZ. Against *S. aureus* clinical isolates the reported MIC values were 5.0 and 10 mM, however cinnamaldehyde was found to inhibit biofilm formation at concentration five times higher than the MIC [128]. Cinnamaldehyde (**25**) showed an MIC value of 358 µM against *Fusarium verticillioides*, the phytopathogenic fungi infecting maize that can also produce mycotoxins during the storage of grains [129]. Treatment of *F. verticillioides* with the aldehyde produced morphological alterations of the hyphae and the cell wall, which resulted in cytoplasmic leakage. Cinnamaldehyde showed weak antifungal activity against *C. albicans* and *Candida tropicalis* with MIC values of 3.0 and 3.8 mM respectively [130], however lower MIC values have been reported [131,132]. Against wood decay fungi, *Laetiporus betulina* and *Laetiporus sulphureus* the potency was comparable, with MIC values around 750 µM [133,134].

From the medicinal plant *Piper taiwanense*, caffeic aldehyde (**133**) (Figure 6) was isolated and found to have significant antitubercular properties (MIC_H37Rv_ = 154 µM) [135]. The other natural brothers of cinnamaldehyde, coniferaldehyde (**134**) and sinapaldehyde (**135**) displayed moderate anticandidal activity [136]. More than 60 strains and clinical *Candida* isolates were evaluated for their susceptibility to the three aldehydes. Sinapaldehyde was found to exert the most potent effect with a MIC values between 480 and 960 µM, while the MIC value of coniferaldehyde and cinnamaldehyde varied between 914 µM and 1.83 mM, and between 1.14 and 3.78 mM respectively [136]. Phenotypic characterization of *C. albicans* treated with sub-inhibitory concentrations of the aldehydes showed alterations in cell morphology and the cell wall, as well as damage to the plasma membrane resulting in cell lysis and cytoplasmic leakage. The study suggested inhibition of membrane ATPases, by examination of H^+^ efflux rates in presence of the aldehydes and known ATPase inhibitors [136]. Both coniferaldehyde (**134**) and sinapaldehyde (**135**) showed growth inhibitory properties against three species of *Streptococcus* with MIC ranging from 150 µM to 1.4 mM, being **135** more active against *S. pyogenes* and *S. mutans*, while **134** was more active against *S. mitis* [137].

Interestingly the most active antifungal compound present in cinnamon was identified to be 2-methoxycinnamaldehyde (**136**), achieving MIC values as low as 19 µM against *Microsporum canis* [138], a fungi causing ringworms in pets and tinea capitis in humans. This aldehyde was also active against other fungi including *C. albicans*, with an MIC value of 308 µM, but was however less active against *A. niger* and different species of bacteria [138]. The positional isomer 4-methoxycinnamaldehyde (**137**) was less active with MIC values of 770 µM against *C. albicans* MTCC 3017, *Issatchenkia orientalis* MTCC 231, *P. aeruginosa* MTCC 424 and *Trichophyton rubrum* MTCC 296, and higher values (MIC > 3 mM) against other microorganisms [139]. Clearly, as observed for the cinnamic acids, the substitution on position 2 of the cinnamic skeleton, confer an increase in the antimicrobial effect. The presence of another methoxy groups in position 3, did not provide a substantial effect, as 3,4-dimethoxycinnamaldehyde (**138**) showed MIC values higher than 2.5 mM for all the evaluated microorganisms (Table 4), except against *T. rubrum* MTCC 296 (MIC = 160 µM) [139]. The aldehyde having three methoxyl groups, 3,4,6-trimethoxycinnamaldehyde (**139**), showed a similar profile of activity, being a weak growth inhibitor of species of *Aspergillus*, *Candida*, *Issatchenkia* and *Micrococcus* (MIC > 1.4 mM) but being active against the dermatophyte *T. rubrum* MTCC 296 with an MIC value of 280 µM [139]. The compound 3,4-methylenedioxycinnamaldehyde (**140**) showed to be a potent inhibitor of *T. rubrum* fungi (MIC = 40 µM), with moderate activity against *A. sydowii*, *C. albicans*, *I. orientalis* and *P. aeruginosa* with an MIC value of 350 µM [139]. The aldehyde was less active against other bacteria and fungi. The replacement of a methoxy group in position 4 of **138** for an ethoxy group, as in 3-methoxy-4-ethoxycinnamaldehyde (**141**), slightly decreased the MIC values for most of the compounds, however the specificity against *T. rubrum* and *I. orientalis* decreased substantially. Introduction of a bromide to **138**, as in 2-bromo-3,4-dimethoxycinnamaldehyde (**142**) decreased the antifungal activity against *T. rubrum*, increasing the MIC value to 460 µM [139]. The compound *N*,*N*-dimethyl-4-aminocinnamaldehyde (**143**) was the only tested cinnamaldehyde having an amino group, and displayed an MIC of 180 µM against *T. rubrum*, but was inactive against the bacteria *Enterobacter cloacae* and *Micrococcus luteus* (MIC > 11 mM). The cinnamaldehyde having a nitro substituent 2,2-nitro-4-methylcinnamaldehyde (**144**) showed a similar antimicrobial profile as the other cinnamaldehydes, with a potent growth inhibitory effect against *T. rubrum* (MIC = 160 µM) but with low activity against several species of bacteria (MIC > 2.5 mM) [139].

Cinnamyl alcohol (**145**) is a component of several essential oils, particularly those of *Cinnamomum* species and other Lauraceae plants [140,141,142]. Cinnamyl alcohol showed little inhibitory effect against all the evaluated bacterial and fungal species (Table 4) [12,126,143]. The study of Barber *et al.* published in 2000, reported the antimicrobial activities of 4-coumaryl alcohol (**146**), coniferyl alcohol (**147**) and sinapyl alcohol (**148**) [68]. Comparatively the alcohols were found to be less active than their corresponding aldehydes, showing MIC values equal or higher than 8.0 mM against bacteria and yeasts. Cinnamyl benzoate (**149**) showed little activity against Gram-negative and Gram-positive bacteria (MIC > 10 mM), however it was higly active against dermatophyte fungi *Trycophyton sp*. and *Epidermophyton floccosum* IFO 9045 (MIC = 20 µM) [143]. Two acetate esters of coumaryl alcohol displayed growth inhibition of some fungi and *S. aureus* [144]. 4-Coumaryl acetate (**150**) displayed MIC values higher than 5 mM against *C. albicans* and other fungi, but was however more active against a vancomycin intermediate *S. aureus* (VISA) with an MIC value of 203 µM [144]. The diester 4-coumaryl diacetate (**151**) showed higher anti-fungal activity in comparison with **150**, and additionally the diacetate demonstrated significant antimycobacterial activity with an MIC value of 215 µM against *Mycobacterium smegmatis* mc^2^-155 [145].

**Table 4:** Minimum inhibitory concentration values of cinnamic aldehydes, alcohols and their derivatives (**132**–**151**).

Compound	Microorganism Strain	MIC	Refs.
Cinnamaldehyde (**132**)	*Aspergillus niger* MTCC 404	1.89 mM	[139]
	*Aspergillus sydowii* MTCC 4335	3.78 mM	[139]
	*Aspergillus parasiticus* MTCC 2797	3.78 mM	[139]
	*Bacillus cereus* ATCC 11778	3.2 mM	[127]
	*Bacillus subtilis*	7.6 mM	[124]
	*Bacillus subtilis* MTCC 121	1.89 mM	[139]
	*Burkholderia cepacea* MTCC 438	1.89 mM	[139]
	*Candida albicans* ATCC 2091	1.1 mM	[131]
	*Candida albicans* ATCC 90028	459 µM	[132]
	*Candida albicans* MTCC 3017	470 µM	[139]
	*Candida albicans* STD-1141	3.0 mM	[130]
	*Candida tropicalis* STD-1118	3.8 mM	[130]
	*Clostridium difficile* PHTCC 107	605 µM	[13]
	*Enterobacter cloacae* MTCC 509	1.89 mM	[139]
	*Enterococcus faecalis*	1.89 mM	[12]
	*Escherichia coli*	3.78 mM	[124]
	*Escherichia coli*	3.78 mM	[12]
	*Escherichia coli* ATCC 11105	3.78 mM	[125]
	*Escherichia coli* CGMCC 1.487	2.0 mM	[123]
	*Escherichia coli* MTCC 43	1.89 mM	[139]
	*Escherichia coli* NCIM-2089	7.6 µM	[121]
	*Escherichia coli* NTCT 8196	< 1.5 µM	[122]
	*Escherichia coli* O157:H7	1.89 mM	[125]
	*Fusarium verticillioides*	358 µM	[129]
	*Gloeophyllum trabeum* BCRC 31614	2.3 mM	[134]
	*Helicobacter pylori* ATCC 26695	15 µM	[121]
	*Issatchenkia orientalis* MTCC 231	470 µM	[139]
	*Klebsiella pneumoniae* MTCC 109	3.78 mM	[139]
	*Laetiporus betulina*	750 µM	[133]
	*Legionella pneumophila* JCM 7571	620 µM	[126]
	*Lenzites betulina* BCRC 35296	757 µM	[134]
	*Laetiporus sulphureus*	700 µM	[133]
	*Laetiporus sulphurous* BCRC 35305	757 µM	[134]
	*Malassezia furfur* IP305	757 µM	[131]
	*Micrococcus luteus* MTCC 2470	3.78 mM	[139]
	*Pseudomonas aeruginosa*	7.57 mM	[12]
	*Pseudomonas aeruginosa* MTCC 424	1.89 mM	[139]
	*Pseudomonas aeruginosa* NCTC 9027	10.6 µM	[122]
	*Sclerotinia sclerotiorum*	1.94 mM	[146]
	*Staphylococcus aureus*	1.89 mM	[12]
	*Staphylococcus aureus* MTCC 121	1.89 mM	[139]
	Methicillin-resistant *Staphylococcus aureus*	1.89 mM	[124]
	*Tametes versicolor* BCRC 35253	2.3 mM	[134]
	*Trychophyton rubrum* MTCC 296	470 µM	[139]
Caffeic aldehyde (**133**)	*Mycobacterium tuberculosis* H_37_Rv	154 µM	[135]
Coniferaldehyde (**134**)	*Streptococcus mitis* ATCC 49456T	351 µM	[137]
	*Streptococcus mutans* DMST 26095	1.4 mM	[137]
	*Streptococcus pyogenes* DMST 17020	351 µM	[137]
Sinapaldehyde (**135**)	*Streptococcus mitis* ATCC 49456T	601 µM	[137]
	*Streptococcus mutans* DMST 26095	601 µM	[137]
	*Streptococcus pyogenes* DMST 17020	150 µM	[137]
2-Methoxycinnamaldehyde (**136**)	*Aspergillus fumigatus* Kuboyama	617 µM	[138]
	*Aspergillus niger* stA-2	1.2 mM	[138]
	*Candida albicans* stT-1	308 µM	[138]
	*Cryptococcus neoformans* stY-8	77 µM	[138]
	*Escherichia coli* E-2602	>1.2 mM	[138]
	*Microsporum canis* stT-6	19 µM	[138]
	*Salmonella typhimurium* 75-276	>1.2 mM	[138]
	*Staphylococcus aureus* 209P	1.2 mM	[138]
4-Methoxycinnamaldehyde (**137**)	*Aspergillus niger* MTCC 404	3.08 mM	[139]
	*Aspergillus sydowii* MTCC 4335	3.08 mM	[139]
	*Aspergillus parasiticus* MTCC 2797	3.08 mM	[139]
	*Bacillus subtilis* MTCC 121	6.16 mM	[139]
	*Candida albicans* MTCC 3017	770 µM	[139]
	*Enterobacter cloacae* MTCC 509	3.08 mM	[139]
	*Escherichia coli* MTCC 43	3.08 mM	[139]
	*Issatchenkia orientalis* MTCC 231	770 µM	[139]
	*Klebsiella pneumoniae* MTCC 109	3.08 mM	[139]
	*Micrococcus luteus* MTCC 2470	3.08 mM	[139]
	*Pseudomonas aeruginosa* MTCC 424	770 µM	[139]
	*Staphylococcus aureus* MTCC 121	3.08 mM	[139]
	*Trychophyton rubrum* MTCC 296	770 µM	[139]
3,4-dimethoxy-cinnamaldehyde (**138**)	*Aspergillus niger* MTCC 404	5.20 mM	[139]
	*Aspergillus sydowii* MTCC 4335	5.20 mM	[139]
	*Aspergillus parasiticus* MTCC 2797	2.60 mM	[139]
	*Bacillus subtilis* MTCC 121	5.20 mM	[139]
	*Burkholderia cepacea* MTCC 438	5.20 mM	[139]
	*Candida albicans* MTCC 3017	2.60 mM	[139]
	*Enterobacter cloacae* MTCC 509	10.4 mM	[139]
	*Escherichia coli* MTCC 43	10.4 mM	[139]
	*Issatchenkia orientalis* MTCC 231	2.60 mM	[139]
	*Klebsiella pneumoniae* MTCC 109	10.4 mM	[139]
	*Micrococcus luteus* MTCC 2470	2.60 mM	[139]
	*Pseudomonas aeruginosa* MTCC 424	2.60 mM	[139]
	*Staphylococcus aureus* MTCC 121	5.20 mM	[139]
	*Trychophyton rubrum* MTCC 296	160 µM	[139]
3,4,6-trimethoxy-cinnamaldehyde (**139**)	*Aspergillus sydowii* MTCC 4335	4.50 mM	[139]
	*Candida albicans* MTCC 3017	8.99 mM	[139]
	*Issatchenkia orientalis* MTCC 231	8.99 mM	[139]
	*Micrococcus luteus* MTCC 2470	2.25 mM	[139]
	*Trychophyton rubrum* MTCC 296	280 µM	[139]
3,4-methylenedioxy-cinnamaldehyde (**140**)	*Aspergillus niger* MTCC 404	1.42 mM	[139]
	*Aspergillus sydowii* MTCC 4335	350 µM	[139]
	*Aspergillus parasiticus* MTCC 2797	710 µM	[139]
	*Bacillus subtilis* MTCC 121	2.84 mM	[139]
	*Burkholderia cepacea* MTCC 438	710 µM	[139]
	*Candida albicans* MTCC 3017	350 µM	[139]
	*Enterobacter cloacae* MTCC 509	1.42 mM	[139]
	*Escherichia coli* MTCC 43	2.84 mM	[139]
	*Issatchenkia orientalis* MTCC 231	350 µM	[139]
	*Klebsiella pneumoniae* MTCC 109	1.42 mM	[139]
	*Micrococcus luteus* MTCC 2470	2.84 mM	[139]
	*Pseudomonas aeruginosa* MTCC 424	350 µM	[139]
	*Staphylococcus aureus* MTCC 121	2.84 mM	[139]
	*Trychophyton rubrum* MTCC 296	40 µM	[139]
3-methoxy-4-ethoxy-cinnamaldehyde (**141**)	*Aspergillus niger* MTCC 404	4.85 mM	[139]
	*Aspergillus sydowii* MTCC 4335	4.85 mM	[139]
	*Aspergillus parasiticus* MTCC 2797	2.42 mM	[139]
	*Bacillus subtilis* MTCC 121	4.85 mM	[139]
	*Burkholderia cepacea* MTCC 438	4.85 mM	[139]
	*Candida albicans* MTCC 3017	2.42 mM	[139]
	*Enterobacter cloacae* MTCC 509	9.70 mM	[139]
	*Escherichia coli* MTCC 43	9.70 mM	[139]
	*Issatchenkia orientalis* MTCC 231	4.85 mM	[139]
	*Klebsiella pneumoniae* MTCC 109	9.70 mM	[139]
	*Micrococcus luteus* MTCC 2470	4.85 mM	[139]
	*Pseudomonas aeruginosa* MTCC 424	4.85 mM	[139]
	*Staphylococcus aureus* MTCC 121	2.42 mM	[139]
	*Trychophyton rubrum* MTCC 296	600 µM	[139]
2-bromo-3,4-dimethoxy-cinnamaldehyde (**142**)	*Trychophyton rubrum* MTCC 296	460 µM	[139]
*N*,*N*-dimethyl-4-amino-cinnamaldehyde (**143**)	*Enterobacter cloacae* MTCC 509	11.4 mM	[139]
	*Micrococcus luteus* MTCC 2470	11.4 mM	[139]
	*Trychophyton rubrum* MTCC 296	180 µM	[139]
2-nitro-4-methyl-cinnamaldehyde (**144**)	*Bacillus subtilis* MTCC 121	2.61 mM	[139]
	*Burkholderia cepacea* MTCC 438	2.61 mM	[139]
	*Enterobacter cloacae* MTCC 509	10.5 mM	[139]
	*Escherichia coli* MTCC 43	2.61 mM	[139]
	*Klebsiella pneumoniae* MTCC 109	10.5 mM	[139]
	*Micrococcus luteus* MTCC 2470	2.61 mM	[139]
	*Pseudomonas aeruginosa* MTCC 424	2.61 mM	[139]
	*Staphylococcus aureus* MTCC 121	10.5 mM	[139]
	*Trychophyton rubrum* MTCC 296	160 µM	[139]
Cinnamyl alcohol (**145**)	*Aspergillus niger* 101	7 mM	[143]
	*Aspergillus oryzae* 102	8 mM	[143]
	*Bacillus subtilis* IFO 13721	10 mM	[143]
	*Candida albicans* IFO 0597	7 mM	[143]
	*Epidermophyton floccosum* IFO 9045	3 mM	[143]
	*Escherichia coli*	7.5 mM	[12]
	*Escherichia coli* IFO 3545	9 mM	[143]
	*Enterococcus faecalis*	7.5 mM	[12]
	*Klebsiella pneumonia* IFO 13541	10 mM	[143]
	*Legionella pneumophila* JCM 7571	7.6 mM	[126]
	*Pseudomonas aeruginosa*	7.5 mM	[12]
	*Rhizopus sp.* 103	3 mM	[143]
	*Saccharomyces cerevisiae* Kyokai no. 8	8 mM	[143]
	*Staphylococcus aureus*	3.7 mM	[12]
	*Trichophyton rubrum* IFO 9185	4 mM	[143]
	*Trichophyton violaceum* IFO 31064	4 mM	[143]
4-Coumaryl alcohol (**146**)	*Bacillus subtilis* 8649	8.0 mM	[68]
	*Escherichia coli* 12210	>8.0 mM	[68]
	*Pseudomonas syringae* 649	>8.0 mM	[68]
	*Saccharomyces cerevisiae* 019391	>8.0 mM	[68]
	*Schizosaccharomyces pombe* 039917	>8.0 mM	[68]
	*Sporobolomyces roseus* 043529	8.0 mM	[68]
Coniferyl alcohol (**147**)	*Bacillus subtilis* 8649	>8.0 mM	[68]
	*Escherichia coli* 12210	>8.0 mM	[68]
	*Pseudomonas syringae* 649	>8.0 mM	[68]
	*Saccharomyces cerevisiae* 019391	>8.0 mM	[68]
	*Schizosaccharomyces pombe* 039917	>8.0 mM	[68]
	*Sporobolomyces roseus* 043529	>8.0 mM	[68]
Sinapyl alcohol (**148**)	*Bacillus subtilis* 8649	>8.0 mM	[68]
	*Escherichia coli* 12210	>8.0 mM	[68]
	*Pseudomonas syringae* 649	>8.0 mM	[68]
	*Saccharomyces cerevisiae* 019391	>8.0 mM	[68]
	*Schizosaccharomyces pombe* 039917	>8.0 mM	[68]
	*Sporobolomyces roseus* 043529	>8.0 mM	[68]
Cinnamyl benzoate (**149**)	*Aspergillus niger* 101	>10 mM	[143]
	*Aspergillus oryzae* 102	>10 mM	[143]
	*Bacillus subtilis* IFO 13721	>10 mM	[143]
	*Candida albicans* IFO 0597	>10 mM	[143]
	*Epidermophyton floccosum* IFO 9045	20 µM	[143]
	*Escherichia coli* IFO 3545	>10 mM	[143]
	*Klebsiella pneumoniae* IFO 13541	>10 mM	[143]
	*Rhizopus sp.* 103	>10 mM	[143]
	*Saccharomyces cerevisiae* Kyokai no. 8	>10 mM	[143]
	*Trichophyton rubrum* IFO 9185	20 µM	[143]
	*Trichophyton violaceum* IFO 31064	20 µM	[143]
4-coumaryl acetate (**150**)	*Candida albicans* ATCC 10231	5.2 mM	[144]
	*Microsporum canis* ATCC 36299	10.4 mM	[144]
	*Staphylococcus aureus* ATCC 25923	6.5 mM	[144]
	*Staphylococcus aureus* VISA 24	203 µM	[144]
	*Tricophyton rubrum* ATCC 28188	10.4 mM	[144]
4-coumaryl diacetate (**151**)	*Candida albicans* ATCC 10231	2.7 mM	[144]
	*Microsporum canis* ATCC 36299	2.7 mM	[144]
	*Mycobacterium smegmatis* mc^2^ 155	215 µM	[145]
	*Staphylococcus aureus* ATCC 25923	2.7 mM	[144]
	*Staphylococcus aureus* VISA 24	672 µM	[144]
	*Tricophyton rubrum* ATCC 28188	2.7 mM	[144]

## 5. Synthetic Cinnamic Hybrids

The interest in making hybrids molecules result from the idea of combining the biological properties of two active molecules to yield a third molecule (chimera or mermaid) with a stronger effect [147]. The conjugation can be used to modulate selectivity, spectrum of activity, potency, and physicochemical parameters. Theoretically the hybrid molecule once administered to biological systems could be lysed to yield *in situ* the two active molecules with atomic economy, or the hybrid molecule could be made sterically flexible enough to act on the two domains of the biological targets as a single piece [148]. Several cinnamic hybrids have been prepared [149,150,151,152,153] but only a few of them have been evaluated for their antimicrobial properties.

The studied cephem hybrids **152**–**155** (Figure 6 and Table 5) are cephalosporin moieties linked covalently to a 2,5-dichlorocinnamic (Figure 4). These hybrids showed a potent growth inhibitory effect against *Staphylococcus epidermidis* A24548, *Staphylococcus haemolyticus* A21638 and *S. pneumoniae* A28272 with MIC values in the nanomolar range [154]. The cephem **155** having a cinnamic aldehyde and a 2-hydroxy-3-propylamine *N*-substitution on the pyridine ring, was the most active against *S. epidermidis* A24548 (MIC = 22 nM) and *S. haemolyticus* A21638 (MIC = 87 nM). The oxazolidinone hybrid (**156**) showed strong antibacterial activity against *E. faecalis* ATCC 29212 (MIC = 194 nM), *Enterococcus faecium* ATTC 700221 (MIC = 388 nM) and *S. aureus* ATCC 29213 (MIC = 194 nM) [155]. Against the methicillin-resistant *S. aureus* ATCC 33591, the cinnamic hybrid (**156**) displayed an MIC value of 1.6 µM. A peptide hybrid based on the human cysteine protease inhibitor cystatin C has been prepared and evaluated for its antimicrobial properties [156]. The peptide cystapep 1 (**157**), having a cinnamic acid residue, showed MIC values of 25.2 µM against *S. aureus* and *Streptococcus pyogenes* and slightly higher MIC values against other *Streptococcus* species. This is the only antimicrobial peptide cinnamic hybrid known so far. The rifamycin hybrid SV (T9) (**158**) (Figure 6) having a cinnamic acid residue linked through amide bond with the piperazine ring, is slightly more potent than rifampin (MIC_T9_ = 106 nM *vs.* MIC_RIF_ = 243 nM) [157]. The molecule has demonstrated to be useful in clinical application [158]. The MIC values of the hybrid molecule were in the nanomolar range against the pathogenic mycobacteria *Mycobacterium avium* complex, *Mycobacterium leprae* and *M. tuberculosis* [157,159,160]. However the hybrid T9 is not active against rifampin-resistant *M. tuberculosis* strains.

**Figure 7:**
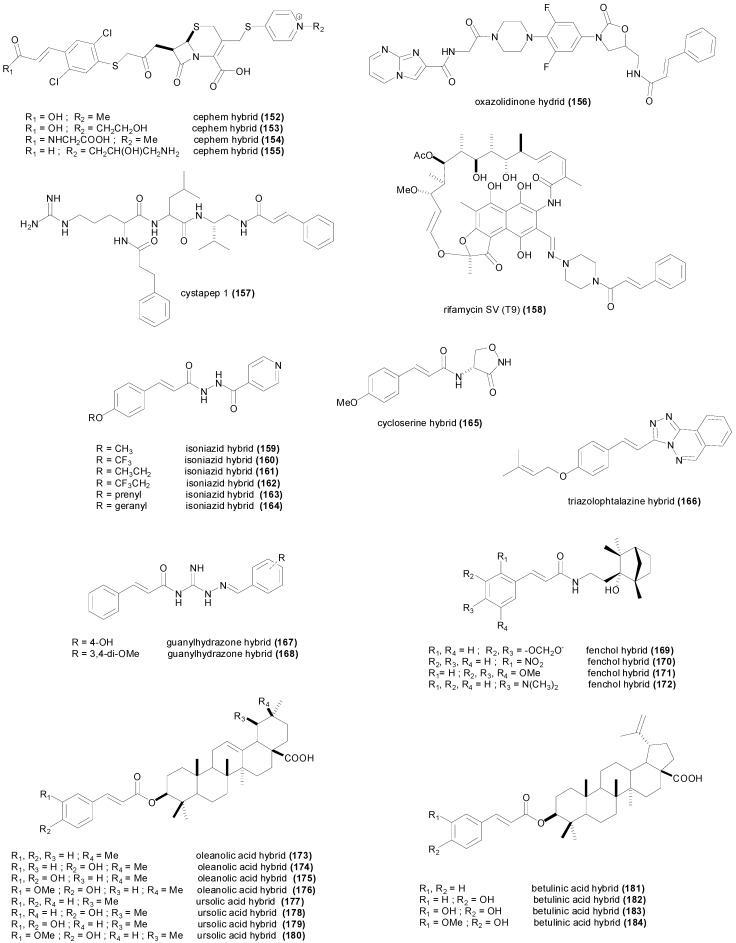
Chemical structures of the cinnamic hybrids **152**–**184**.

Cinnamic acid hybrids have also been prepared with other antitubercular drugs including isoniazid **159**–**164** and cycloserine (**165**) (Figure 7).

The isoniazid 4-methoxycinnamoyl hybrid (**159**) was the most active (MIC = 300 nM) among the screened hybrids [161], but was however slightly less active than isoniazid itself (MIC = 182 nM) [162]. The presence of larger substituents on the 4-*O*-position, decreased anti-TB activity.

Among the prepared 4-coumaric hybrids from the same comprehensive study, the cycloserine hybrid **165** displayed an MIC value of 950 µM [161], while the literature MIC value of cycloserine is 245 µM [161,163]. Nonetheless the study identified the triazolophtalazine cinnamic derivative **166** with a significant MIC value of 1.4 µM against the H_37_Rv strain and a selectivity index around 320 in comparison to THP-1 cells [161]. Guanyl hydrazone hybrids have also been prepared and examined for antimycobacterial activity [164]. Hydroxy substitution of the benzaldehyde hydrazone increased the anti-TB activity, with the hybrid **167** having a hydroxyl in position 4 displaying an MIC value of 40.5 µM against the virulent H_37_Rv strain [164]. The presence of 3,4-dimethoxy substitution on the benzaldehyde hydrazone, as in the hybrid **168**, increased growth inhibition (MIC = 8.9 µM).

Thirty-one fenchol hybrids were prepared with different substitutions on the cinnamic ring, and four hybrids **169**–**172** resulted with potent activity against *M. tuberculosis* H_37_Rv [165]. The fenchol hybrids **169** and **170** having respectively a 3,4-methylenedioxy and a 2-nitro substituents (**170**) on the aryl ring of the cinnamic moiety, displayed an MIC value of 6.7 µM (Table 5). The fenchol hybrid **171** with 3,4,5-trimethoxy substitution on the cinnamic acid showed higher potency (MIC = 2.4 µM), while the hybrid **172** having 4-dimethylamine substitution was the most active (MIC = 540 nM) against the H_37_Rv pathogenic strain [165].

**Table 5:** Minimum inhibitory concentration values of cinnamic hybrids (**152**–**184**).

Compound	Microorganism Strain	MIC	Refs.
cephem (**152**)	*Streptococcus pneumoniae* A28272	200 nM	[154]
	*Enterococcus faecalis* A20688	1.6 µM	[154]
	*Staphylococcus aureus* MRSA A27223	3.2 µM	[154]
	*Staphylococcus epidermidis* A24548	95 nM	[154]
	*Staphylococcus haemolyticus* A21638	200 nM	[154]
cephem (**153**)	*Streptococcus pneumoniae* A28272	760 nM	[154]
	*Enterococcus faecalis* A20688	1.5 µM	[154]
	*Staphylococcus aureus* MRSA A27223	3.0 µM	[154]
	*Staphylococcus epidermidis* A24548	91 nM	[154]
	*Staphylococcus haemolyticus* A21638	190 nM	[154]
cephem (**154**)	*Streptococcus pneumoniae* A28272	350 nM	[154]
	*Enterococcus faecalis* A20688	1.4 mM	[154]
	*Staphylococcus aureus* MRSA A27223	2.8 mM	[154]
	*Staphylococcus epidermidis* A24548	42 nM	[154]
	*Staphylococcus haemolyticus* A21638	175 nM	[154]
cephem (**155**)	*Streptococcus pneumoniae* A28272	727 nM	[154]
	*Enterococcus faecalis* A20688	364 nM	[154]
	*Staphylococcus aureus* MRSA A27223	364 nM	[154]
	*Staphylococcus epidermidis* A24548	22 nM	[154]
	*Staphylococcus haemolyticus* A21638	87 nM	[154]
oxazolidinone hybrid (**156**)	*Enterococcus faecalis* ATCC 29212	194 nM	[155]
	*Enterococcus faecium* ATTC 700221 (VRE)	388 nM	[155]
	*Staphylococcus aureus* ATCC 29213	194 nM	[155]
	*Staphylococcus aureus* ATCC 33591 (MRSA)	1.6 µM	[155]
cystapep 1 (**157**)	*Staphylococcus aureus* ATCC 29213	25.2 µM	[156]
	*Streptococcus agalactiae* NTCC 8181	50.5 µM	[156]
	*Streptococcus anginosus* CCUG 27298	50.5 µM	[156]
	*Streptococcus pneumoniae* ATCC 49619	50.5 µM	[156]
	*Streptococcus pyogenes* type M1	25.2 µM	[156]
rifamycin T9 (**158**)	*Mycobacterium avium* 101	15.9 nM	[159]
	*Mycobacterium avium* N-260	831 nM	[157]
	*Mycobacterium tuberculosis* H_37_Rv	31.9 nM	[159]
	*Mycobacterium tuberculosis* H_37_Rv	106 nM	[157]
	*Mycobacterium tuberculosis* MTB9 (RIF-R)	8.5 µM	[159]
isoniazid hybrid (**159**)	*Mycobacterium tuberculosis* H_37_Rv	300 nM	[161]
isoniazid hybrid (**160**)	*Mycobacterium tuberculosis* H_37_Rv	1.1 µM	[161]
isoniazid hybrid (**161**)	*Mycobacterium tuberculosis* H_37_Rv	1.3 µM	[161]
isoniazid hybrid (**162**)	*Mycobacterium tuberculosis* H_37_Rv	2.2 µM	[161]
isoniazid hybrid (**163**)	*Mycobacterium tuberculosis* H_37_Rv	2.3 µM	[161]
isoniazid hybrid (**164**)	*Mycobacterium tuberculosis* H_37_Rv	1.9 µM	[161]
cycloserine hybrid (**165**)	*Mycobacterium tuberculosis* H_37_Rv	950 µM	[161]
triazophtalazine hybrid (**166**)	*Mycobacterium tuberculosis* H_37_Rv	1.4 µM	[161]
guanylhydrazone hybrid (**167**)	*Mycobacterium tuberculosis* H_37_Rv	40.5 µM	[164]
guanylhydrazone hybrid (**168**)	*Mycobacterium tuberculosis* H_37_Rv	8.9 µM	[164]
Fenchol hybrid (**169**)	*Mycobacterium tuberculosis* H_37_Rv	6.7 µM	[165]
Fenchol hybrid (**170**)	*Mycobacterium tuberculosis* H_37_Rv	6.7 µM	[165]
Fenchol hybrid (**171**)	*Mycobacterium tuberculosis* H_37_Rv	2.4 µM	[165]
Fenchol hybrid (**172**)	*Mycobacterium tuberculosis* H_37_Rv	540 nM	[165]
oleanolic acid hybrid (**173**)	*Mycobacterium tuberculosis* H_37_Rv	85.2 µM	[166]
oleanolic acid hybrid (**174**)	*Mycobacterium tuberculosis* H_37_Rv	10.4 µM	[166]
oleanolic acid hybrid (**175**)	*Mycobacterium tuberculosis* H_37_Rv	323 µM	[166]
oleanolic acid hybrid (**176**)	*Mycobacterium tuberculosis* H_37_Rv	19.8 µM	[166]
ursolic acid hybrid (**177**)	*Mycobacterium tuberculosis* H_37_Rv	>341 µM	[166]
ursolic acid hybrid (**178**)	*Mycobacterium tuberculosis* H_37_Rv	10.4 µM	[166]
ursolic acid hybrid (**179**)	*Mycobacterium tuberculosis* H_37_Rv	323 µM	[166]
ursolic acid hybrid (**180**)	*Mycobacterium tuberculosis* H_37_Rv	4.95 µM	[166]
betulinic acid hybrid (**181**)	*Mycobacterium tuberculosis* H_37_Rv	>341 µM	[166]
betulinic acid hybrid (**182**)	*Mycobacterium tuberculosis* H_37_Rv	10.4 µM	[166]
betulinic acid hybrid (**183**)	*Mycobacterium tuberculosis* H_37_Rv	323 µM	[166]
betulinic acid hybrid (**184**)	*Mycobacterium tuberculosis* H_37_Rv	316 µM	[166]

In a study from 2008, three triterpenes, betulinic, oleanolic and ursolic acids, were esterified in the 3-hydroxyl position, with different cinnamic acids with the aim of generating novel molecules which could potentially inhibit the *M. tuberculosis* H_37_Rv bacteria [166]. The cinnamic acids employed for the preparation of the esters were cinnamic, 4-coumaric, caffeic and ferulic acids. The esters having the 4-coumaroyl moiety, 3-*O*-(4'-coumaroyl) oleanolic acid (**174**), 3-*O*-(4'-coumaroyl) ursolic acid (**178**) and 3-*O*-(4'-coumaroyl) betulinic acid (**182**) were among the esters with the highest anti-TB activity, achieving MIC values of 10.4 µM. The MIC values of the free triterpenoids were 109 µM for oleanolic and betulinic acids, and 27.4 µM for ursolic acid [166], and therefore conjugation with 4-coumaric acid increased the anti-TB potency. Moreover the ester with the highest activity, 3-*O*-feruloyl ursolic acid (**180**), was able to inhibit completely the growth of *M. tuberculosis* at a concentration of 4.95 µM. The cinnamate and caffeate esters showed moderate to little anti-TB activity, with MIC values higher than those obtained for the free triterpenoids.

## 6. Conclusions

This review summarizes the *in vitro* antimicrobial activity of several cinnamic-related molecules by collating the reported MICs in a comprehensive list. Because the MIC data included in this review was extracted from several studies (using different experimental methods), it is far from ideal to compare the MIC as absolute values, but rather the MICs should be used as relative numbers indicating the tendency of the compounds to inhibit certain microorganisms. This review primarily serves as a framework to quickly identify the cinnamic acids and related molecules that have been tested for their antimicrobial properties.

Among the cinnamic-related molecules with the highest antimicrobial activity, the hybrids between antibiotics and cinnamic acids, such as the rifamycin T9 (**158**) and the oxazolidinone **156** with MIC values in the nanomolar range, were the champions. However the activity of these hybrids is mostly due to the potent effect of the antibiotic component. It is unknown whether the conjugates actually hydrolyze *in vivo* to yield two active molecules with potential synergism, or it is the whole molecule responsible for the observed biological effect. Among the non-hybrid cinnamic-related molecules with the lowest MIC values 4-methoxycinnamic acid (**9**), 3-nitrocinnamic acid (**11**), all the caffeoyl quinic acids **25**–**30**, most of the cinnamate and 4-coumarate esters **40**–**61**, and most of the cinnamoyl and 4-coumaroyl amides **78**–**115** are worth mentioning. A remarkable antimicrobial activity was detected for isobutyl cinnamate (**45**) achieving a broad spectrum of activity against yeasts, Gram-positive and Gram-negative bacteria, with MIC values between 43 and 12 µM. Further studies need to confirm the potent antimicrobial activity observed, expanding the screening to other microorganisms and drug resistant-isolates, and finally evaluating its toxicity. This example illustrate how a simple substitution, for instance comparing isobutyl cinnamate (**45**) with butyl cinnamate (**44**), can have a significant impact on the biological properties of the molecules (4-fold MIC change against some microorganisms).

There is no doubt that the cinnamic acids, their derivatives and hybrid molecules display marked antimicrobial effects. Some microorganisms are more sensitive to a chemical class than others. For instance, fungal organisms are generally more susceptible to the cinnamic aldehydes, while the cinnamic acids, esters and amides tend to affect more importantly the bacteria. A noteworthy effect was observed for the cinnamic molecules against *Mycobacterium tuberculosis*. The growth of the TB-causing bacteria was repeatedly inhibited by micromolar concentrations of molecules containing the cinnamic acid moiety. However very little is known about the mechanism of action of cinnamic acid, and the essential structural features required for anti-TB activity. Detailed molecular studies of the biological targets of the cinnamic acids may help to design high-affinity cinnamic-based ligands which may be important for developing future therapeutic alternatives to the growing problem of drug-resistant microbial pathogens.
